# Molecular dynamics modeling the synthetic and biological polymers interactions pre-studied via docking: Anchors modified polyanions interference with the HIV-1 fusion mediator

**DOI:** 10.1007/s10822-014-9749-8

**Published:** 2014-05-27

**Authors:** Vladimir B. Tsvetkov, Alexander V. Serbin

**Affiliations:** 1 Biomodulators and Drugs Research Center, Health RDF, Adm. Ushakova 14-209, 117042 Moscow, Russia; 2 Topchiev Institute of Petrochemical Synthesis, RAS, Leninsky Pr. 29, 119991 Moscow, Russia; 3 Institute for Physical-Chemical Medicine, Malaya Pirogovskaya Str. 1a, 119828 Moscow, Russia

**Keywords:** Molecular dynamics, Docking, Polymer–biopolymer interaction, Drug design, Maleic acid copolymer, Norbornane/tetracyclododecen derivatives, HIV entry (fusion) inhibitor, Glycoprotein gp41

## Abstract

In previous works we reported the design, synthesis and in vitro evaluations of synthetic anionic polymers modified by alicyclic pendant groups (hydrophobic anchors), as a novel class of inhibitors of the human immunodeficiency virus type 1 (**HIV-1**) entry into human cells. Recently, these synthetic polymers interactions with key mediator of HIV-1 entry-fusion, the tri-helix core of the first heptad repeat regions [**HR1**]_3_ of viral envelope protein gp41, were pre-studied via docking in terms of newly formulated algorithm for stepwise approximation from fragments of polymeric backbone and side-group models toward real polymeric chains. In the present article the docking results were verified under molecular dynamics (**MD**) modeling. In contrast with limited capabilities of the docking, the MD allowed of using much more large models of the polymeric ligands, considering flexibility of both ligand and target simultaneously. Among the synthesized polymers the dinorbornen anchors containing alternating copolymers of maleic acid were selected as the most representative ligands (possessing the top anti-HIV activity in vitro in correlation with the highest binding energy in the docking). To verify the probability of binding of the polymers with the [HR1]_3_ in the sites defined via docking, various starting positions of polymer chains were tried. The MD simulations confirmed the main docking-predicted priority for binding sites, and possibilities for axial and belting modes of the ligands–target interactions. Some newly MD-discovered aspects of the ligand’s backbone and anchor units dynamic cooperation in binding the viral target clarify mechanisms of the synthetic polymers anti-HIV activity and drug resistance prevention.

**Electronic supplementary material:**

The online version of this article (doi:10.1007/s10822-014-9749-8) contains supplementary material, which is available to authorized users.

## Introduction

A complementary development of docking and molecular dynamics (**MD**) techniques in application to a computer-aided modeling of specific interactions between synthetic and biological polymers is very promising platform for novel advancements in modern medicine, biotechnology and pharmacy. Particularly, in antiviral drug design area, the computational modeling of synthetic polymers interference with glycoprotein gp41 mediators of the human immunodeficiency virus type 1 (**HIV-1**) entry into human cells may open new prospects toward preventive anti-HIV inhibitors. Such drugs are vitally needed for modern anti-HIV/AIDS^1^ prophylaxis and therapy [1, 2].

**Scheme 1:**
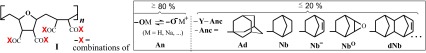
The observable synthetic polymers. **An**—Anionic side groups; **Anc**—pendant anchors derived from hydrophobic alicycles: adamantane (**Ad**), norbornane (**Nb**), norbornen (**Nb** ^**=**^), epoxynorbornane (**Nb** ^**O**^), dinobornene (**dNb**)—i.e., tetracyclo-[4.4.0.1^2.5^1^7.10^]-dodecene, etc.; **Y**—spacer/bridge fragment linked the Anc with polymeric backbone chain

In previous works we reported the design, synthesis and in vitro^2^ evaluations of the novel series of anti-HIV inhibitors based on synthetic anionic polymers modified by alicyclic pendant groups (hydrophobic anchors) through the variable spacers (bridges) [3–8]. Within this series of polymers the alternating cyclocopolymers of divinyl ether with maleic acid/their salt (see Scheme 1, formula **I)** possess the amplified anti-HIV activity [9], when the side groups (X = OH/ONa) are partially (optimally, the 6–8 % of total amount of the X-groups) substituted by the cage-type anchors (**Anc**).

**Fig. 1:**
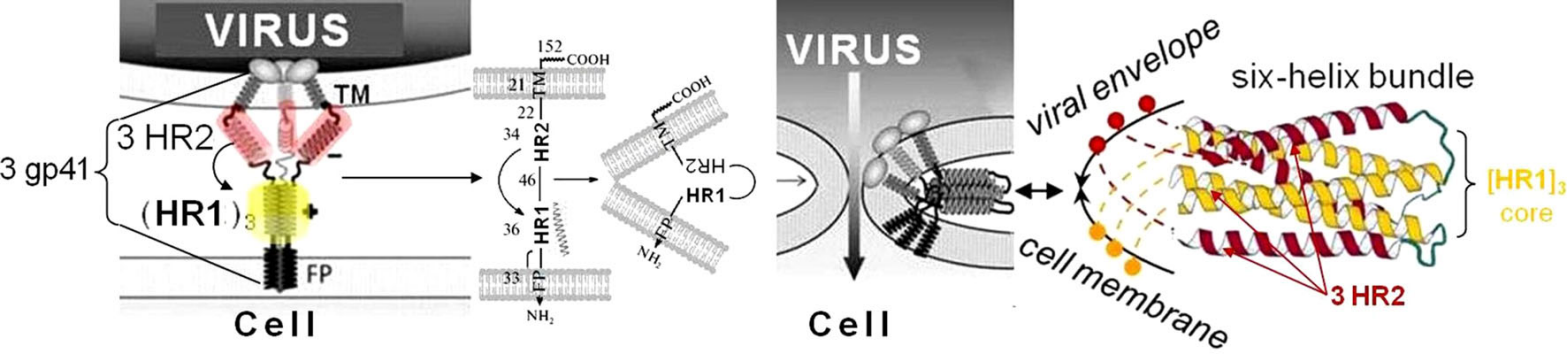
HIV-1 entry. Adsorption of HIV-1 virion on a permissive cellular membrane (with CD4 and CCR5/CXCR4 receptors) exposing the three gp41 molecules. Their hydrophobic N-terminus (FP)_3_ are anchored in lipid matrix of cell membrane, while the C-tails (3 TM) are bounded in the virus envelope. Ectodomains between the FP and TM came into self-aggregation, forming tri-helix cationic intermediate (3 HR1 → [HR1]_3_) that becomes a core for consecutive hairpin-like folding with three helices of contra-charged (anionic) second heptad repeat regions (3 HR2). The resulted collapsing leads to the fusion, which can be prevented via therapeutic blocking the [HR1]_3_ core against contacts with the HR2 helixes [11–15]

It was found that these polymeric compounds affect the earliest stage of the HIV-1 entry. Partially retarding the HIV-1 virions adsorption on cell membrane, they were able to block the next (post-adsorption) step, fully preventing the virus penetration through cell membrane toward cytoplasm and nucleus [3, 7]. Therefore, reviewing [7–10] the HIV-1 entry, we concentrated upon the post-adsorption event, namely, the fusion. It is well known [11–15] that the fusion is driven by the HIV-1 envelope glycoprotein gp41. Within the gp41 our attention was focused on the gp41 ectodomain first heptad repeat region (**HR1**), the amino acid sequence Arg542–Leu581. Three molecular self-assembly of these regions results in the three-helix complex (3 HR1 → [HR1]_3_), the key intermediate of the HIV-1 envelope fusion with a permissive cell membrane (Fig. 1).

The polymers of formula **I** we originally predicted and designed for therapeutic intervention against the [HR1]_3_ complex, as potentially most sensitive target in the view of both electrostatic and hydrophobic selectivity criteria [7]. The [HR1]_3_ possesses the combination of positively charged and hydrophobic sites that, in theory, should be attractive for the synthetic polymers (Scheme 1) complementarity through anions (An) and anchors (Anc) simultaneously. Therefore, just the [HR1]_3_ we taken into consideration as the most probable target for the polymers I attack.

Then this assumption has been supported by results of a computer-aided modeling the interactions between these polymeric compounds and the viral biopolymer target via the docking. The docking was performed recently in terms of newly formulated algorithm for step-by-step approximation from fragments of polymeric backbone and side-groups toward real polymeric chains [9, 16]. Among sub-domains of the gp41 ectodomain (Fig. 1) the [HR1]_3_ complex was identified exactly as the target capable of powerful binding with the models of formula **I** synthetic polymers. And the binding energies of variable side-groups (X) combinations were in good correlation with the in vitro experimental data [9, 16].

**Fig. 2:**
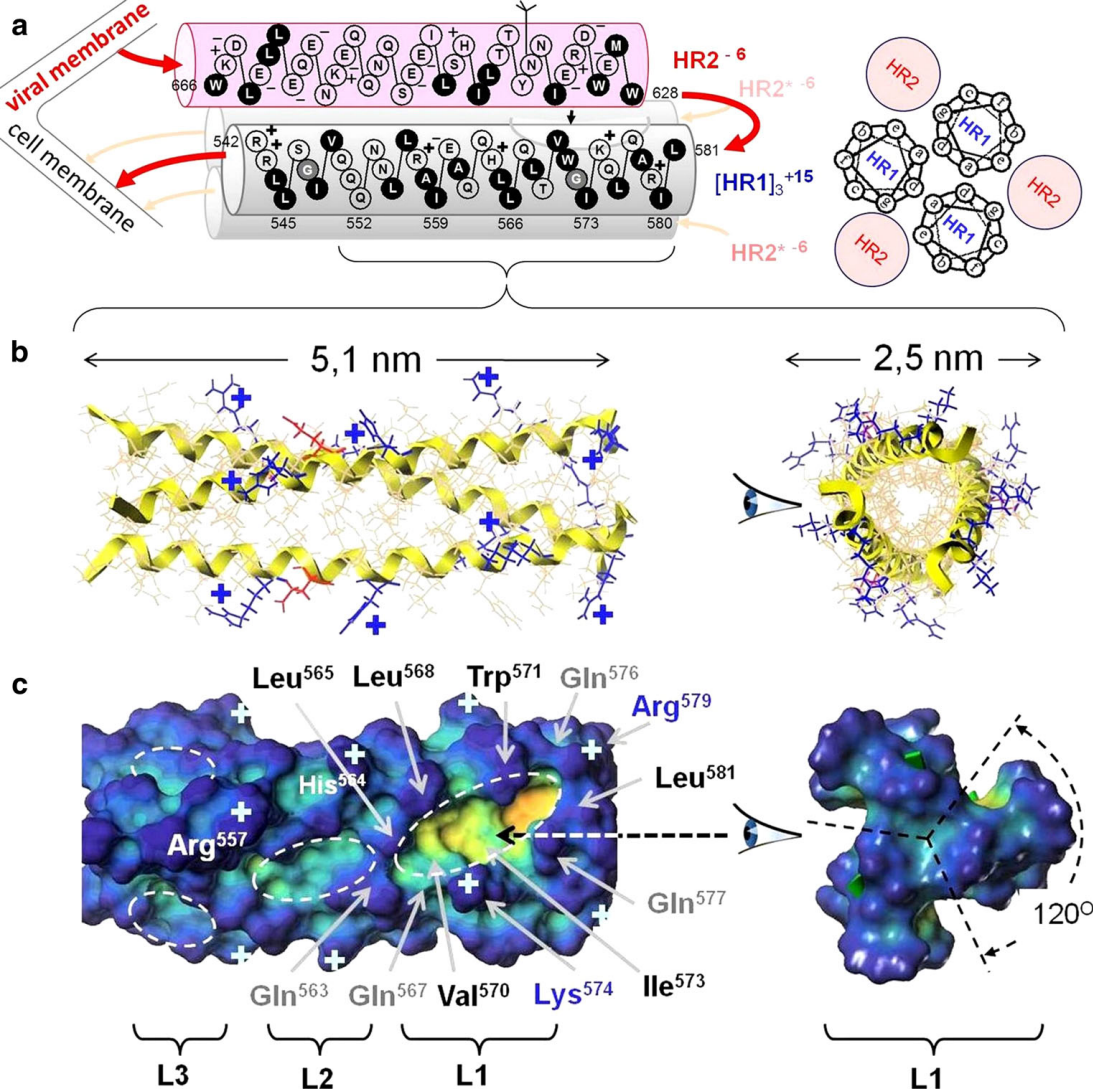
3D organization of the [HR1]_3_ complex, a target for the anti-HIV-1 fusion intervention. **a** Scheme of amino acid sequences and resulted excesses of electric charge for both HR1 and HR2 (at right the connection between the three HR2 and three HR1 coiled-coil core is shown in view from C-tail for the HRI α-helixes repeat heptad motifs order: *… a*, *b*, *c*, *d*, *e*, *f*, *g…*). **b**, **c** The [HR1]_3_ core model, simulated from 1AIK database and displayed in ribbon and depth-gradation forms, respectively. In the ribbon the basic amino acids are labeled by **+**. In **c** three levels (the *L1*, *L2*, and *L3*) of cavity triplets (where every cavity repeats itself three times between adjacent α-helices on the every level) are represented. Amino acids surrounding one among triplet of deepest cavities (so-called “pockets”) within *L1* are shown in detail. The *L1* pocket and smaller cavities of *L2/L3* areas are outlined with a dotted curve

Within the [HR1]_3_ 3D structure (Fig. 2) the triplets of cavities/pockets between adjacent parallel α-helices were observed around the target at least on the three levels: **L1** (Gln567–Arg579), **L2** (Leu556–Leu565) and **L3** (Val549–Asn553). Altogether there were nine hollows which could provide stable contacts with ligands. All these cavities were identified by the docking as zones of intensive binding the polymers’ units from both anionic backbone^3^ and hydrophobic anchors insertable into the cavities. The L1 pockets, being the deepest cavities, were the most powerful binding locus for majority of the docking-tested models of the polymer **I** motives [9, 16]. Similar findings were reported by many other drug design investigators focused a docking-based computer-aided screening the HIV-1 fusion inhibitors within the L1 pockets [17–22]. Such focusing only to the L1 triplet of pockets without any consideration of additional capacities of the L2 and L3 triplets of cavities could be acceptable just in case of small molecule ligands. The size of small molecules (typically ≤1 nm) cannot cover simultaneously more than one cavity/pocket on the one from among L1/L2/L3 levels, where the L1 is preferable in the ligand-binding competition with the L2/L3.

However, as soon as we deal with the polymeric compounds, the mentioned situation is converted cardinally: the polymeric chain of extended length (comparable with the nano-scale of the biopolymeric target) provides the extended possibilities for simultaneous multipoint contacts with more than one, several or full-scale binding vacancies on the target’s surface. Just such tendency to polyvalent binding has been discovered by the docking pre-study of the polymers **I** in connection with the [HR1]_3_ complex [9, 16].

**Fig. 3:**
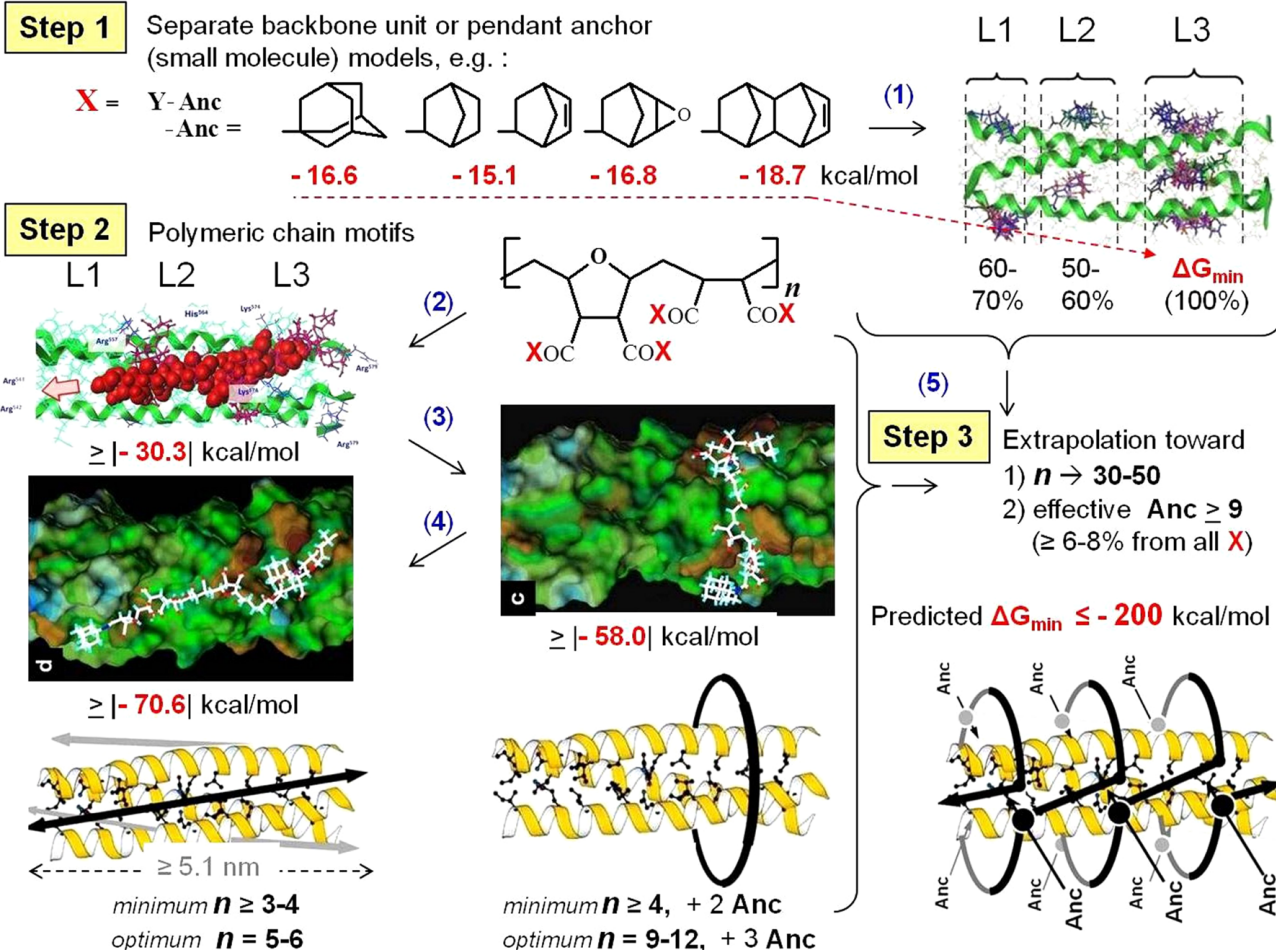
Three-step docking-based modeling the polymers I–[HR1]_3_ interactions, the main results [9, 16]. *Step 1* Small molecule size models of the polymer’s fragments, e.g. (*1*), bind the target within the three levels (*L1*–*L3*) of triplet cavities around target, preferably the deepest pockets at the *L1*, where the models of Anc possess maximal binding energies |−15.1| to |−18.7| kcal/mol (the dNb is the most active Anc). *Step 2* oligomeric models of the polymeric chain motifs provides more effective binding with capacity of both axial and belting orientation, depending on absence (*2*) or presence (*3*–*4*) of active Anc and distances between the Anc along the polymeric chain. *Step 3* An extrapolation of the *steps 1–2* results toward the *real size of polymers* ***I*** *chains,* predicting possibility for significant combined binding, ΔG_bind_ ≥ |−200| kcal/mol

It was found [9, 16] that, depending on the degree of polymerization (***n***), absence/presence of hydrophobic anchors (among the side-groups X) and distances between these anchors, the models of formula **I** (Scheme 1) were capable of binding the [HR1]_3_ by different modes (Fig. 3).

*The first mode* (**A**) *is an axial connection* of the ligand chains along the viral target helixes. *The alternative mode* (**B**) *is belting* the target (due to the anchors contacts with hydrophobic cavities within one level, predominantly, the L1. *The third mode* (**AB**) represents *an integrative combined axial*-*co*-*belting binding* the target by full-scale polymeric chains (in theoretical extrapolation toward the real anti-HIV active polymeric compounds of formula **I** in Scheme 1, where the degree of polymerization is ***n*** ≥ 30 with content of anchors ≥9, i.e., the 6–8 % among the X-groups).

Thus the computer-aided modeling of polymers **I**–[HR1]_3_ interactions via docking^4^ leaded to very useful preliminary information, clarifying probable molecular mechanisms of the synthetic polymers intervention in HIV-1 entry (fusion). From the other hand, the docking procedure have some fundamental limitations: (1) the docking is limited by size of ligand models and directly can be applied exclusively for relatively short fragments of the real polymeric chains; (2) it takes into account conformational flexibility of ligands only, while the target is considered in general as a crystal-like rigid structure.^5^ All these restrictions can be overcome by **MD**.

Briefly resuming the docking results, in the current article we consider the MD-based verification of the main docking-predicted binding sites/modes of interactions between the viral target and synthetic polymers of formula **I**. The obtained from MD results are compared with the docking ones. In addition, new light on peculiarities of dynamic interactions between the synthetic and biological polymers is discussed in prospect for novel drug design development.

## Experimental

The suggested methodology for stepwise docking, applied early to pre-studying the considered polymeric objects, was described in our previous reports [9, 16] in details. The same *viral target* model, based on 1AIK PDB [23] in application to the trimeric complex [HR1]_3_, was used for the MD simulation as the starting target’s 3D-conformation. This model consists of triplet of the 36 amino acid α-helixes (within the HR1 repeat heptad motifs) self-assembled in the coiled-coil represented in Fig. 2.

*The models for analyzed synthetic polymers (of the formula* **I** *)* were built by means of SYBYL 8.0 molecular graphics software package (Tripos Inc., St. Louis, USA). In view of the big size of polymeric models, partial charges on their atoms were determined by the Gasteiger-Hückel method [24]. The MD simulations were performed by using a suite of programs Amber 9 [25].

*Modeling of the polymer folding into coil* was carried out in implicit solvent. The use of implicit solvent was realized with application of Hawkins-Cramer-Truhlar (HCT) model [26] within Generalized Born/Solvent-Accessible Surface Area (GB/SA) formalism [27] in the presence of 0.1 M NaCl. The General Amber Force Field (GAFF) [28] was utilized for calculating interatomic interaction energy between the polymer atoms. At the beginning of MD simulations in implicit solvent (GB–MD), the models energy was minimized using 250 steps of the steepest descent followed by 250 steps of conjugate gradient. Then gradual heating to 300 K during 20 ps was performed. To avoid wild fluctuations into the studied systems at this stage, weak harmonic restrains were used with a force constant of 5 kcal × mol^−1^×Å^−2^ for all atoms, excepting hydrogen. The SHAKE algorithm [29] was applied to constrain the bonds to hydrogen atoms that allowed using a 2 fs step. Dielectric constants of 1 (interior) and 80 (exterior) were employed in GB–MD simulations. The production phase of GB–MD simulations was carried out until the radius of gyration of the coil wasn’t counterbalanced (about 10 ns). Then such conformation of a coil, which corresponded to an energy minimum, was chosen for further researches. To control the temperature, the Langevin thermostat with the collision frequency of 1 ps^−1^ was used.

Interactions between the synthetic polymers and the gp41-derived target were investigated with account of solvent influence, taking the solvent in an explicit form. The explicit (TIP3P) [30] solvent simulations were performed in a cubic water box with periodic boundary conditions imposed. To calculate interatomic interaction energy the necessary parameters for all atoms were taken from the force fields ff03 [31] and GAFF [28] mentioned above. The negative charges were neutralized by adding Na^+^ ions randomly. The complexes were minimized prior to the simulations. Firstly, the locations of the solvent molecules (and ions) were optimized for 1,000 steps (500 steps of a steepest descent minimization followed by 500 steps of a conjugate gradient minimization) with all the solute atoms, being restrained to their positions with a force constant of 500 kcal × mol^−1^×Å^−2^. Then, the complex structure was optimized without any restriction for 2,500 steps (1,000 steps of steepest decent followed by 1,500 steps of conjugate gradient). Subsequently, a gradual heating to 300 K over 20 ps was performed. To avoid wild fluctuations in the system at this stage, weak harmonic restraints with a force constant of 10 kcal × mol^−1^×Å^−2^ were used for all atoms with the exception of the solvent atoms. As well as in a case of the solvent in an implicit form all bonds to hydrogen atoms were constrained using the SHAKE algorithm, which allowed a time step of 2 fs. The non-bound 1–4 van der Waals and electrostatic interactions were scaled by standard Amber values (SCEE = 1.2, SCNB = 2.0). The cutoff for van der Waals interactions was set to 12 Å during minimization and heating, and 10 Å during following MD simulations. Long-range electrostatics was calculated using the particle mesh Ewald method [32]. The MD simulations in a production phase were performed at *p* = 1 atm by using the Berendsen barostat with isotropic position scaling (NTP) = 1, compressibility of the system (COMP) = 44.6 (default), pressure relaxation time (TAUP) = 2 ps, and keeping the T = 300 K under the control of the Langevin thermostat with a collision frequency of 1 ps^−1^. The trajectory length was 80 ns. Snapshot visualization was performed using VMD [33, 34]. The snapshots were taken every 0.1 ns. For *hydrogen (H) bond* identification and analysis the following criteria of the H bonds recognition and approval were applied: a donor–acceptor distance of 3.5 Å and an angle cutoff of 30°.

### MM–GBSA scoring energy of MD simulation

In this investigation the MM–GBSA method was used to calculate the binding free energy of complex state. Within this approach, the binding free energy of a complex was calculated in accordance with the formula (1):

1 $$ \Delta G_{bind} = G_{complex} {-}G_{target} {-}G_{ligand} $$

where *G*_*target*_, *G*_*ligand*_ and *G*_*complex*_ were the energies of the target ([HR1]_3_ complex), ligand (model of polymer **I**) and complex (between the target and the ligand), respectively.

The free energy of complex oneself and its each component were scored by the following formula (2):

2 $$ G = E_{MM} + G_{sol} {-}TS $$

where *E*_*MM*_, *G*_*sol*_ and *TS* were total mechanical energy of the molecule in gas, the free energy of hydration and entropic contribution, respectively. *E*_*MM*_ was calculated as the sum of electrostatic, van der Waals energies and energy of internal strain (bonds, angles and dihedrals) by using a molecular-mechanics approach. *G*_*sol*_ was calculated as the sum of polar *G*_*polar*_ and nonpolar *G*_*nonpolar*_ terms, where the electrostatic contribution to the hydration energy *G*_*polar*_ was computed with applying a GB approach [27] through the algorithm developed by A. Onufriev, D. Bashford and D. A. Case (OBC) [35, 36] for calculating effective Born radii. The nonpolar component of hydration energy *G*_*nonpolar*_ (including a solute–solvent van der Waals interaction and energy required for forming cavity being equal of solute volume in solvent) was calculated by using the formula: *G*_*nonpolar*_ = *α* × *SASA*, where SASA was solvent accessible surface area computed with application of LCPO method [37], and *α* was set to 0.00542 kcal mol^−1^ Å^−2^. Because of very high conformational capacity of the polymeric ligand, it was difficult to score a contribution to entropy change at the expense of change of conformational mobility. Therefore it was decided not to consider the change of entropy in free energy. It was suggested also that change of internal energy of the target and the ligand contributed to the energy of binding much weakly than other kinds of energy, and therefore, this change was excluded from a consideration as well. For each system snapshots taken from a single trajectory of the complex, the MD simulation was used for the calculations of the binding free energy. Dielectric constants for calculating in gas-phase and water-phase were set to 1 and 80, respectively.

For an estimation of contacts of a polymer (as a whole) and its separate components with a target the following definition of the “contact” was applied: a contact of (fragment of) polymer with target should be considered as the contact taken place, if at least anyone couple atoms (including the hydrogen) appeared so that one of the atoms belong to the target, and another to the (fragment of) polymer, and if the distance between these atoms became less or equal to some critical value d. In this studying the d was taken equal 3 Å.

## Results and discussion

### Polymeric ligands’ models in various starting positions to viral target

**Scheme 2:**
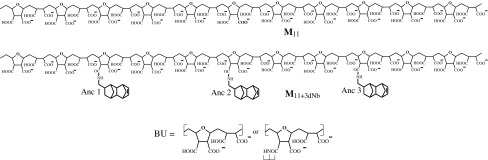
The anchor-free (M_11_) and anchor-containing (M_11+3dNb_) fragments of polymeric chains. *Note* In contrast with the pendant anchors (Anc) every single anionic structural unit repeated itself (11 times) along the polymeric chain backbone we designed as the BU

Taking into account the previous docking results (and their correlation with in vitro anti-HIV evaluations data), the models M_11_ and M_11+3dNb_ (Scheme 2) were studied as the representative fragments of anchor-free and anchor-containing polymeric chains, respectively.

The first model (without pendant anchors) is related to moderately anti-HIV-1 active kind of the polymeric compounds **I** (X = OH/ONa, 100 %), whereas the second model simulates the most active structure among the compounds **I** (X = OH/ONa, 92–94 %, and dNb, 6–8 %), possessing the top anti-HIV activity in vitro [7, 9] in good correlation with the highest binding energy in the docking [9, 16]. In accordance with the docking results-based prediction (Fig. 3, Step 3) the eleven monomers (***n*** = 11) in the polymeric chain of the tried models should be enough for both axial (***n*** = 5–6) and one-level belting (***n*** = 9–12) modes of binding the target. And in case of the **M**_11+3dNb_ the existence of exactly three active (dNb) anchors provide the docking-prognosticated possibility of the modeled molecular structure to occupy either three-level (L1–L3) cavities along the target’s α-helix (axial connection) or triplet cavities around the every level (belting).

**Fig. 4:**
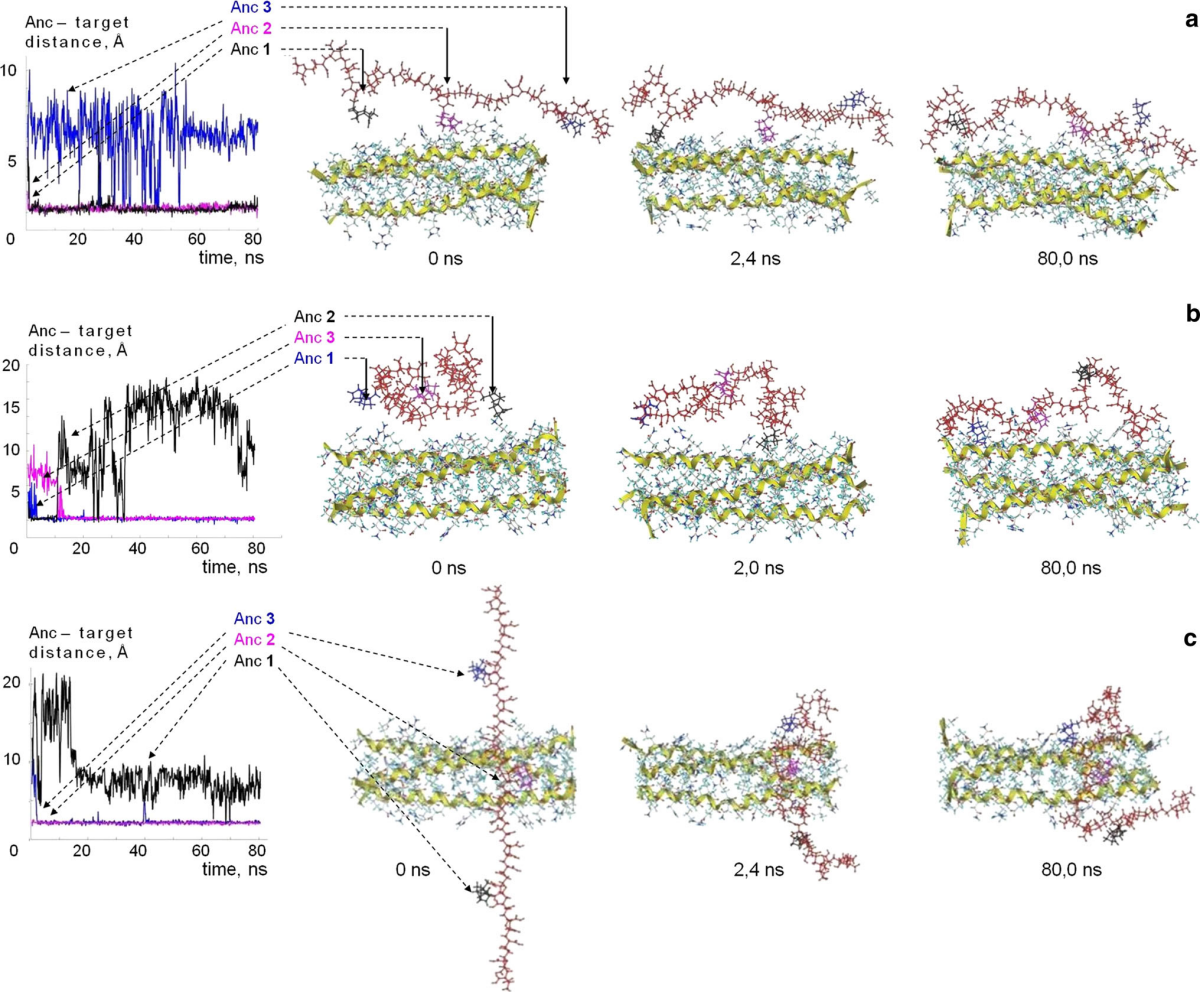
MD-simulated interactions between the M_11+3dNb_ and the [HR1]_3_ target for the different starting positions: **a** SP1, **b** SP2, and **c** SP3. On the *left* a dynamics of every anchor–target distances, and on the *right* snapshots

To verify the binding sites and ligands’ orientations defined via docking, the MD modeling was performed, using various starting positions (**SP**) of the polymeric chain. Besides, the SP variations were tested to find whether the modes of binding between the ligands and target depended on the SP conditions as well as to estimate a comparative efficiency of the different possible modes [38]. The first type of SP (**SP1**) was the polymeric chain linearly unfolded along the target’s α-helix, using the **M**_11_ or **M**_11+3dNb_ models. In the second type of start (**SP2**) we dealt with the three anchors containing model **M**_11+3dNb_, pre-folded in coil that contacted with the target near the one of α-helixes. And in the third type of the start (**SP3**) we tested the unfolded polymeric chain **M**_11+3dNb_ oriented transversely to the target. The MD simulated development of the positions SP1, SP2 and SP3, illustrated by some snapshots within the simulated time (80 ns), are shown in Fig. 4a–c, respectively.

The MD evaluation of the models **M**_11_ (not shown) and **M**_11+3dNb_ (Fig. 4a) from longitudinal (axial) starting position SP1 confirmed evidently the docking-predicted capacity of both anchor-free and anchor-containing polymeric ligands **I** to be effective binding agents in axial direction along the target’s α-helix within all three levels (L1–L3) of cavities. An analogous result was observed if instead of the unfolded chain **M**_11+3dNb_ its coil-type conformation was tested (see Fig. 4b). On the contrary, a transversely oriented starting position of unfolded **M**_11+3dNb_ chain (located near L1 pockets of target) displayed tendency to belt the target intensively covering at least two α-helices exactly at the L1 pockets region (Fig. 4c). The last result well correlates with docking-predicted belting mode of interaction if the polymeric chains **I** are equipped by active pendant anchors (Fig. 3).

An analysis of the ligand–target conformations/snapshots development from the variable starts (Fig. 4)^6^ leaded to statement of the following facts with preliminary conclusions:

1. during the full time of the MD simulations the presence of the synthetic polymers’ models M_11_ or M_11+3dNb_ (as ligands) didn’t disturb main self-organization of the biopolymeric (protein-type) target that preserved a stable three α-helix 3D structure [HR1]_3_ in conformations closely related to one, early tested as a rigid crystal via the docking [16] (Fig. 2); this [HR1]_3_ stability under the MD simulated physiologically relevant temperature confirms, particularly, a validity of the target 3D structure application for the early considered docking-based pre-modeling in respect of the viral target and the tested type of synthetic polymers as ligands;
2. SP1 and SP2 conditions resulted in axial binding along the target, whereas SP3 demonstrated tendency toward belting. Together these computer-aided data obtained by MD from all starting positions (SP1–SP3) well correlated with the docking predicted modes of the target binding (Fig. 3);
3. MD generated conformations were also in good correlation with the docking-determined epicenters of active interactions between the ligands and the target, including the binding priority of the L1 pockets region and additional binding potency of the L2 and L3 cavities too.

Confirming the docking results in general, the MD modeling (in comparison with the docking) opened many new aspects of various specific target-ligands contacts and their multipoint cooperation in view of time evolution and the time-accumulated statistic data. The significant details and tendencies are considered below.

### Dynamic evolution of distances between ligand’s and target’s substructures

Very interesting aspect (undetectable via docking) is evolution of the various intermolecular contacts in dynamics. Particularly, the diagrams of ligand’s Anc(s)–target distances dynamics of the **M**_11+3dNb_ model (Fig. 4) shown that at least two from three Anc(s) achieved stable contacts with target during very short time—by the first 1–3 ns (from the varied starts). And then the slightly fluctuating but enough stable contacts were kept mainly over whole period of MD-simulation (80 ns). This fact indicated that the pendant anchors (linked with polymeric chain through flexible bridges –Y– = –NH–CH_2_–) possessed great mobility for rapid finding vacancies suitable for the binding stabilization on the target’s surface. Such capability of anchors to be quickly contacting sensors of the ligand–target interactions should be taken into special consideration (see below, the section “MD-based revision of the polymeric platform advantages for drug design”).

### Statistics of contacts between structural components of target and ligands

**Fig. 5:**
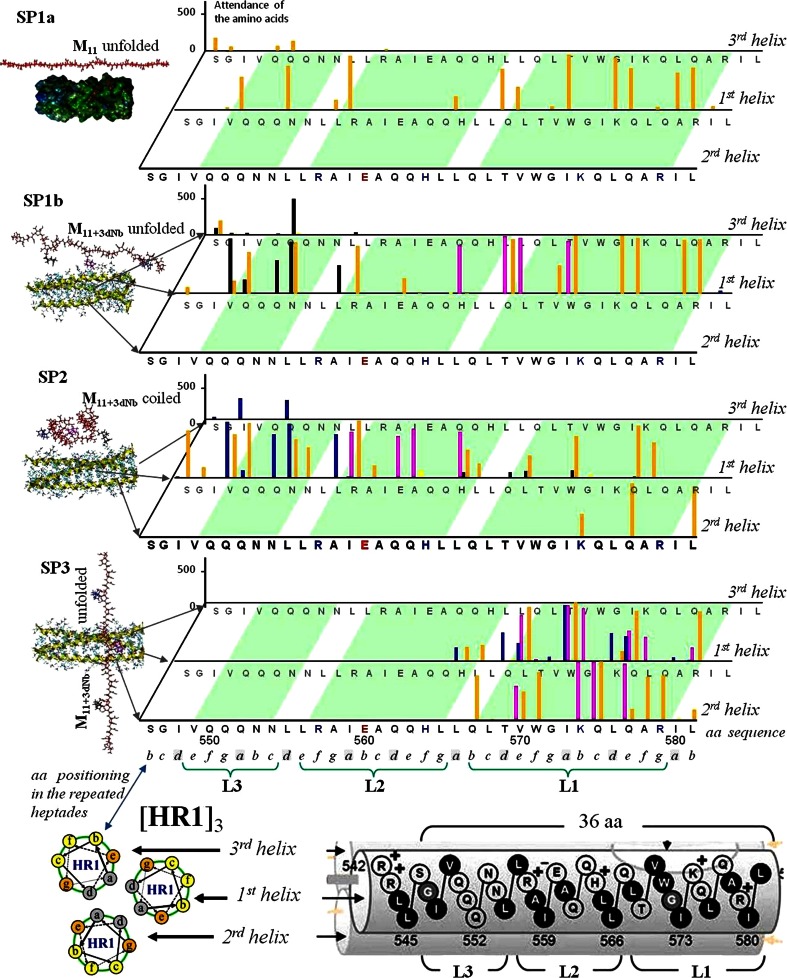
Statistics of the ligand–target contacts within 80 ns in separate consideration of an attendance of every amino acid (aa) among sequence of the [HR1]_3_ target (including the three identical 36 aa α-helixes, formally designed as 1st, 2nd, and 3rd) by various components of the ligands: anionic units of polymeric chain backbone with X = OH/ONa (yellow columns) or hydrophobic pendant anchors (X = dNb), the first Anc (blue columns), second Anc (crimson columns) and third Anc (black columns). **SP1a**—start from Anc-free **M**_11_ unfolded along the 1st helices; **SP1b**—start from similarly oriented Anc-containing **M**_11+3dNb_ (Fig. 4a); **SP2**—the **M**_11+3dNb_ started from a coil connected with target near 1st helix (Fig. 4b); **SP3**—the same ligand model started from unfolded conformation transversally to the target near the L1 floor of main pockets triplet, from the 1st helix side (Fig. 4c). The below shown scheme of the [HR1]_3_ amino acids ordering indicates the heptad repeat positions ***a*** and ***d*** involvement in the coiled-coil self-aggregation, whereas positions ***b***, ***c***, ***e***, ***f*** and ***g*** are accessible to contacts with ligands

More detailed understanding of the ligand-target interaction under various starting conditions can be extracted from a statistical analysis of the MD-simulated multipoint contacts between structural components of the ligand and amino acid residues of the target. The corresponding examples are represented in Fig. 5 and Table 1.

**Table 1:**
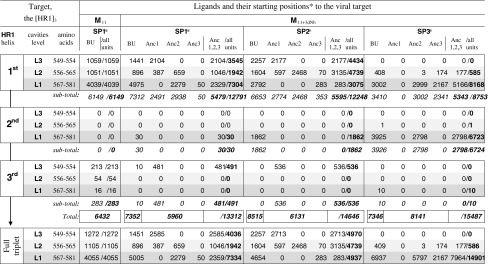
The multiplicity of attendance of the target’s amino acids by ligand’s components: 80 ns-accumulated statistic data of the ligand–target contacts quantity for partial contributions of anionic units of polymeric chain backbone (BU) and each hydrophobic pendant anchor (Anc1, Anc2 and Anc3) of the ligands to connections with the target’s amino acids of the all cavities/pockets levels (L1, L2, L3) of every HR1 helixes

* The various starting positions of ligand to target: SP1—unfolded along the target’s 1st helix, SP2—coiled near the 1st helix and contacted with pockets locus L1 of the target, and SP3—unfolded transversely to the target near its pockets locus L1

The MD-based investigation of quantitative attendance of the target’s amino acid sites by various structural components of the tested ligands leaded to the following observations.

#### Target’s amino acids accessibility depending on their positioning in α-helixes

First of all, we would like attract the attention to differences between the target’s amino acids in relation to variety of their positioning within the repeat heptad motifs (… ***a, b, c, d, e, f, g***,…) of α-helixes and native involvement of amino acids into coiled-coil self-assembly of the viral target. For the both models of ligands their components practically didn’t attend the amino acids in the “***a***” and “***d***” positions (Fig. 5). This tendency is in good agreement with well known [11, 12, 39] involvement of the noted amino acid residues in self-aggregation of the α-helixes together—to the coiled-coil type three-helix complex [HR1]_3_ (Fig. 2). As it was mentioned above, this complex kept stability during the full MD simulations. Therefore, using a binding potentiality for the target’s coiled-coil self-organization, these amino acids (I548, Q552, L555, I559, Q562, L566, T569, I573, L576 and I580) should be restricted for interactions with the external ligands. No intensive attendance of the ***a*** and ***d***—positioned sites by any components of the MD-tested ligands (from all SP) was recorded, for exclusion only the several cases.^7^ In the most considerable cases of Q552 and L576 the both amino acids were located in epicenters of the cavity/pocket (at L3 and L1, respectively). So, an enhanced accessibility of these amino acids to contacts with ligands can be explained by possibilities of ligand’s structures to penetrate into the cavity/pocket toward the polypeptide backbones of the target-forming helixes.

Other amino acids in positions “***b***”, “***c***”, “***e***”, “***f***” and “***g***” of the repeated heptads (Fig. 5) form an interface well accessible for the target–ligands interactions. Total statistic (for M_11_ from SP1, and for M_11+3dNb_ from SP1, SP2 and SP3 cases) resulted in the following partial contributions to the amino acids attendance, depending on positioning in the heptads (% of all contacts): ***a*** (1.3), ***b*** (24.6), ***c*** (18.7), ***d*** (2.2), ***e*** (12.6), ***f*** (29.5) and ***g*** (11.1).

#### Contacting activity of different levels (L1–3) of the target’s cavities/pockets

The ligands–target contacts intensity was different for the different levels L1/L2/L3 of target’s triplets of cavities (Fig. 5, Table 1). The largest—L1 pockets were most attractive for the contacting. They became fully dominant region in interaction with **M**_11+3dNb_, providing 96.2 % of total contacts (Table 1), if the ligand model started from the transversal position located near the L1. Therefore, the MD confirms the docking-predicted role of the L1 pockets of target as a general locus sensitive to the ligands **I** attacks.

However, the other levels (L2 and L3) cavities were capable of substantial contributions to the contacts, when the starting position of ligand didn’t create a favourable precondition for the level L1 attendance. For instance, the start SP2 resulted in nearly equal partial contributions of every level (Table 1). The high potentiality of L2/3 cavities is in a good correlation with data of the docking pre-study as well. The marked activity of L2 and L3 cavities in additional multipoint binding the ligands was predicted by docking on the condition that the ligands were not small molecules (considered by other authors) but the polymeric compounds (tested in our work). It was found that only the poly/oligo-meric compounds, such as **I** (Scheme 1, when n ≥ **4**), were able to cover the full nano-scale (≥5.1 nm) of the all three levels of cavities simultaneously (Fig. 3).

#### Role of the ligands’ anionic chain ([BU]_11_) and hydrophobic pendant anchors (Anc1–3)

The MD-based statistics demonstrated important peculiarities determined by the cooperation of acidic (anionic-ionizable and H-bond capable) polymeric chain backbone units (BU) and side-grafted to the polymeric chain pendant anchors (hydrophobic Anc1, Anc2 and Anc3). An estimation of relative contributions of the both kinds of structural species into the ligand potency to interact with the target was analyzed from comparative study of the fully acidic model **M**_11_ (containing 44 acidic groups, the X = OH/O^−·^Na^+^ 100 %) and the derived model **M**_11+3dNb_, where only 3 from 44 (7 %) side groups were substituted by the anchors. A comparison of the anchor-free with anchor-containing models in their interactions with the viral target (Fig. 5) resulted in statement of the fact: under similar starting position (SP1) the anchor-“equipped” model generated significantly more intensive contacts with the target. From data summarized in Table 1 one can see, that degree of total attendance of target’s amino acids by **M**_11+3dNb_ (13,312 contacts) was more than twice higher than by **M**_11_ (6,432 contacts).

Interestingly, losing the three anionic side-groups (because of them substitution by the hydrophobic pendant anchors) didn’t lead to decreasing a multiplicity of contacts of anionic BU with target: 6,432 contacts of **M**_11_, as compared with 7,352 contacts of **M**_11+3dNb_. The similar enhanced contacting potency of the anionic backbone of **M**_11+3dNb_ was recorded for other starting conditions as well: 8,515 (SP2) and 7,346 (SP3) total contacts of BUs with target (Table 1). Besides, the anchors themselves also strongly contributed to the contacting: 5,960 (SP1b), 6,131 (SP2) and 7346 (SP3) additional contacts with target (ibid).

Inserting the anchors in polymeric ligand altered a general distribution of contacts of the anionic backbone components with target not significantly (compare distribution of yellow columns of SP1a and SP1b, Fig. 5).

Therefore, from the MD statistics-based point of view, the pendant anchors (grafted to polymeric chain) didn’t crucially compete/interfere with the polymeric chain itself (the backbone) for binding the target’s amino-acid sites. Quite the contrary, the anchors promote a synergetic effect, amplifying the degree of BU–target contacts, plus the anchors (Anc1, Anc2 and Anc3) themselves contributed an additional capacity to multipoint connection with the viral target (Fig. 5; Table 1). This MD statistic results are in good agreement with the docking modeling: the anchor containing models provided more powerful binding with the target, as compared with anchor-free models [9, 16]. Moreover, together both MD and docking data correlate very accurately with the data of in vitro evaluations of real polymeric compounds of the series **I** (where exactly the dNb containing experimental samples were ~12-folds more anti-HIV active than their anchor-free precursors [4, 9]).

In sum of all MD-tested SP (Fig. 5) the target’s amino acid residues priority to be attended by BUs and/or Anc1–3 can be classified for the following three groups: (**1**) acids preferably connectable with anionic units of polymeric chain (S546, Q550, R557, R579,^8^ and L581); (**2**) acids more sensitive to pendant anchors (Q551, L556, E560, A561, and G572); and (**3**) “plural” acids actively attended by both polymeric chain’s units and anchors (V549, N553, H564, Q567, L568, V570, W571, K574, Q575, Q577, and A578). As a whole, the activity of these amino acid residues is in a good correlation with docking predicted binding sites. For example, both docking and MD defined identical main sites of active contacts with ligand models: R557 and R579 (preferably with the acidic BU components) and K574, A578, W571, and V570 (with both BU and Anc(s)).

#### Influence of starting ligand-target orientation on the ligand-target contacts distribution

The Fig. 5 visually demonstrates that both anchor-free anionic chain **M**_11_ and anchor containing derivate **M**_11+3dNb_ starts from unfolded state SP1 (axially to the 1^st^ helix of viral target) leaded to intensive contacts with amino acids just of the 1st helix. The anchor-containing model provided more intensive contacts than **M**_11_, v.s.

An analogous statistics of the **M**_11+3dNb_ ligand was observed for the start SP2—from coil state near the middle of the 1st helix. Both unfolded (SP1) and coiled (SP2) starting conformations leaded to similar contacts with the target. The initially unfolded conformation developed toward partial folding, and the coil conformation was in progress toward partial unfolding along the 1st helix. But the both starting conformations resulted in similar situations by the 80 ns of the MD simulation (Fig. 4, snapshots series ***a*** and ***b***, respectively). Within the 80 ns the coiled start SP2 development accumulated a statistics of contacts preferably with the target’s 1st helix (like the SP1 start, but all contacts were distributed between various levels L1–3 and helixes rather more evenly than in the case of SP1 start) (Table 1).

In contrast with the mentioned starts, the conformation of **M**_11+3dNb_ unfolded transversally to the target near L1 didn’t develop contacts with target along the full length of 1st helix. The ligand’s interactions with target were concentrated around the L1 pockets of 1st and 2nd helixes generally (SP3, Fig. 5; Table 1).

The significant influence of starting orientation on a mode of the ligands–target interactions (discovered by the considered MD simulation) could not be found evidently via docking. Clearing this aspect, the MD at the same time confirms the docking predicted possibilities for the sufficiently long chain ligands **I.** The binding capability of both axial (along one helix) and belting (around L1 pockets within at least two helixes) contacts were observed.

### Statistics and dynamics of H-bonds

Hydrogen bonds (H-bonds) are of great importance in molecular biology, and so the H-bonds role in the modeled system of the protein-type target [HR1]_3_ interactions with the synthetic polymeric ligands **I** was taken into special consideration. First of all, this problem was considered in respect of H-bonds network for self-organization of the protein target (intra-stabilization of the coiled-coil [HR1]_3_ complex). After that the target’s capability-implementation for H-bonds formation with external ligands was analyzed in focus for specificity of the polymers series **I.**

#### ***Some definitions applied in this article for the H*-*bonds analysis*** (in detail see *supplementary material 1*)

**Q**_**Hb**_—Quantity of H-bonds within a single snapshot; **D**_**Hb**_—degree of H-bonds formation (H-bonding) statistically averaged for a time interval of MD simulation:

3 $$ D_{Hb} = \varSigma^{m} Q_{Hb}^{i}/{m} $$

where the *Q*_*Hb*_^*i*^—amount of H-bonds for analyzed pool of N/O atoms in *i’th* target/ligand (or target + ligand) conformation (in *i’th* single snapshot), *m*—number of the MD generated conformations (snapshots) taken into account for analysis, and the *Σ* ^*m*^ *Q*_*Hb*_^*i*^—total amount of the H-bonds within the *m* snapshots.

After the calculations for all possible pairs of N and O atoms, these data sets were treated for quantitative estimation of summarized amounts or degrees of H-bonds as well as for analysis of separate contributions of various sub-structural components of ligands and target to the H-bond network. To differentiate various analytically relevant series of H-bonds we introduced the following definitions for H-bonds classification, depending on the H-bond forming atoms belonging: **M/M-**H-bond between atoms from main chain (the polypeptide backbone) of target; **M/S-**H-bond between atom(s) of the main chain and atom(s) of an amino acid residue side chain or **S/M-**H-bond between atom(s) of an amino acid residue side chain and atom(s) of the main chain; **S/S-**H-bond between atoms from the side chains of target; **M/L-**H-bond between atom(s) from target’s main chain and atom(s) of ligand; **S/L-**H-bond between atom(s) from a side chain of target and atom(s) of ligand

#### H-bonds of the target’s self-organization in the coiled-coil [HR1]_3_ complex

An analysis of H-bonds network for the protein target self-organization in presence of the polymeric ligand can be extracted from the data represented in Table 2 by an example of MD simulated [HR1]_3_–M_11+3dNb_ system from start SP1.

**Table 2:**
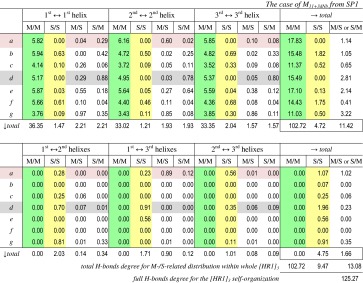
Degree of the H-bonds formation (*D*_*Hb*_) within the [HR1]_3_ complex for intra-α-helix (1st–1st/2nd–2nd/3rd–3rd) and inter-α-helixes (1st–2nd/1st–3rd/2nd–3rd) contacts in view for involving amino acid residues at different heptad repeat positions (*a*–*g*) trough main polypeptide backbone or side-chain atoms (O or N)

*Notes* The columns take into account H-bonds between N/O atoms of main polypeptide backbone (**M**) or of amino acid’s side-chain (**S**) for separate estimation of “main chain ↔ main chain” (**M/M**), “side chain ↔ side chain”(**S/S**) and “main chain ↔ side chain” (**M/S** or **S/M**) H-bonds; the two N-terminal (Ile580 and Leu581) and two C-tail (Ser546 and Gly547) amino acids of every from three α-helixes were excluded from the estimation because of edge effects of the target cutoff (zones of a priori inadequacy between modelled and natural 3D structure of the target)

The first very visible difference of H-bonds organization is observed between the intra- and inter-helix interactions. All H-bonds of *main polypeptide backbone (M/M)* contribute to intra-helix stabilization exclusively (1st–1st/2nd–2nd/3rd–3rd helix). And no M/M H-bonds between different helixes (1st–2nd/1st–3rd/2nd–3rd helixes) were recorded. The amino acid residues order in the M/M H-bonds formation within the each α-helix polypeptide (Ile548-Arg579)^9^ backbone conforms in general with the classic *Pauling*–*Corey*–*Branson alpha helix,* in which every backbone N–H group donates a H-bond to the backbone C=O group of the amino acid four residues earlier (*i* + 4 → *i* H-bonding) [40].

In the MD simulation some dynamically-rearrangement fluctuations of the helix construction were occurred at the physiologically relevant temperature. A total probability of these reversible aberrations from the classical α-helical architecture of H-bonds was ≤11 %. In spite of this, the classic α-helix order for various amino acid residue pairs *i* + 4 → *i* (within the considered 32 amino acid sequences of the [HR1]_3_ helixes triplet) was locally realized by 30–99 %.

Thus, the H-bound potentiality of the target’s polypeptide main chains is mobilized for the α-helix (*4*_*13*_-*helix*) self-formation. It involved the great amount of the M/M-type H-bonds (*D*_*Hb*_ = 102.72), the main part of full intra-[HR1]_3_ H-bonds degree (*D*_*Hb*_ = 125.27), Table 2.

As the polypeptide main chain M/M H-bonds are used in the α-helix intra-organization completely, therefore, this source is excluded from any external inter-helixes or any target-ligand H-bonding. So the external part of H-bonds network requires an involvement of exactly the side chains of α-helixes. Within the concrete amino acid sequence of the target’s α-helixes, only “H-bond competent” N/O atoms in the side chains could be considered as the suitable points. Such amino acid side chain’s capabilities are shown in Table 3 as the frame-marked data.

**Table 3:**
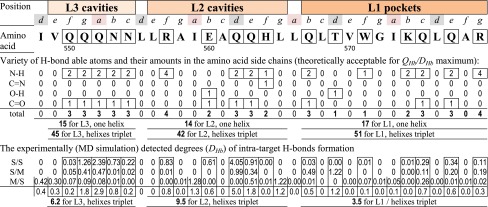
“H-bonds competent” side chains* of 548–579 amino acid residues sequence of every α-helix from the helixes triplet constructed the target [HR1]_3_ complex

* Within full amino acid sequence of single α-helix of target, the amino acids with “H-bonds competent” side chains are marked by frames, and below the contained amount of H-bound competent atomic groups (N–H/C=N/O–H/C=O) as well as degrees of the S/S, S/M and M/S(plus S/M) types of H-bonds are indicated by example of MD simulation of target in presence of M_11+3dNb_ ligand from SP1 start

Thus, the H-bonds capability of the viral target’s helixes is distributed along their polypeptide polymeric chains, depending on chemical nature of amino acid residues, represented in Table 3. The MD statistics estimation resulted in more decreased values of *D*_*Hb*_. But the relative contributions of various amino acids to the S/S H-bonding were in good proportions with the theoretical abilities generally: the most S/S H-bond potential residues provided the main contribution to the H-bonds formation (in the order of R > Q, N > K > W, T, see in Table 3).

However, not only chemical nature but the positioning of the amino acids in the heptad repeat motifs (… ***a, b, c, d, e, f, g*** …) of helixes plays an essential role in the S/S (and S/M or M/S) type H-bonds accomplishment. In the first place the positions ***a*** and ***d*** should be taken into account as theoretically predictable points for selective inter-helix contacts supported the self-aggregation from separate α-helixes toward their complex 3HR1 → [HR1]_3_ (Fig. 2 and 5). Molecular architecture of this viral complex is a special (triplet type) case of the classic coiled-coil self-assembly of α-helixes [41], where the side chains of ***a*** and ***d*** positioned residues are involved into the inter-helical aggregation [11, 12, 39], but not in intra-helix contacts mainly.

Really, under the MD simulation the side chains of amino acid residues at ***a*** and ***d*** positions played no significant role in the intra-helix H-bonding. Simultaneously, just these species made predominant contribution to the S/S type H-bonds between pairs of three helixes co-aggregated together in the [HR1]_3_. The total degree of S/S type H-bonds was very slight (*D*_*Hb*_ = 0.002) in the intra-helix interactions [1st ↔ 1^st^] + [2nd ↔ 2nd] + [3rd ↔ 3rd], just as this value became many times increased (*D*_*Hb*_ = 3.03) within the interactions between adjacent α-helixes [1st–2nd] + [1st–3rd] + [2nd–3rd] (Table 2).

In contrast with the mentioned situation, the side chains of amino acid residues in the heptad repeat positions ***b*** and ***f*** were most active for intra-helixes H-bonding (*D*_*Hb*_ = 3.58), but fully inactive for any H-bonding between the adjacent α-helixes (*D*_*Hb*_ = 0.00) (Table 2).

The next type, the M/S or S/M, of H-bonding between N/O atoms of main polypeptide chain and complemented O/N atoms of a side chain were more preferable for the intra-helix stabilization (*D*_*Hb*_ = 11.42) than for the inter-helixes co-aggregation (*D*_*Hb*_ = 1.66) (Table 2).

Together all considered types of the target own network of H-bonds support the self-stabilization of the HR1 polypeptide chains in the α-helix state (mainly via M/M H-bonds) as well as these α-helixes triplet self-assembly to the coiled-coil [HR1]_3_ complex (using S/S and S/M or M/S H-bonds).

It is very important to note that this complex (as the viral target for therapeutic intervention by the polymeric ligands **I**) was stable at the MD-simulated physiologically relevant temperature in the presence of ligand M_11+3dNb_. Moreover, comparing partial saturation of every α-helixes by the H-bonds (Table 2) with the statistic of the ligand-target contacts (Fig. 5; Table 1), we can conclude that the intensive contacts of the M_11+3dNb_ ligand with the 1st helix didn’t suppress this helix involvement into the H-bonds-mediated self-assembly. The 1st α-helix M/M, S/S and M/S or S/M contributions to the target own H-bonding were no less than (or comparable with) similar contributions of 2nd and 3rd helixes, which were free of active connection with the polymeric ligand under the SP1 start (Table 2). An analogous situation in MD trials of the M_11_ was observed as well.

#### Polymeric ligands capacity of H-bonding for self-stabilization or interaction with target

The alicyclic dNb anchors don’t possess any atoms/groups fit to H-bonding (i.e., the dNb is H-bond inert specie). All H-bond forming potential of the tested models of ligands concentrated in the polymeric backbone [BU]_11_ (due to the 44 carboxy-derived –COX groups plus 11 furan-coupled ether –O– atoms). Both M_11_ and M_11+3dNb_ ligands have enough potent sets of “H-bond competent” sub-structures, allowing degree of H-bonds up to *D*_*Hb*_ = 99, as maximum (Table 4). However, as demonstrated the MD-simulation, only low part of this potency (*D*_*Hb*_ = 4.15–5.44) was used by the ligands for own, intra-molecular, self-stabilization (Table 4).

**Table 4:**
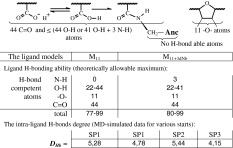
“H-bonds competent” atoms of the polymeric ligands and degree of the intra-ligand H-bonding

**Scheme 3:**
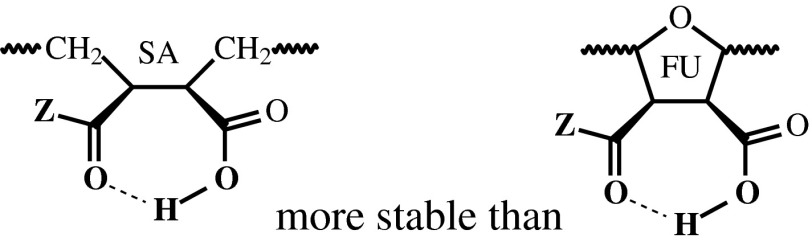
Intra-molecular H-bonds in polymeric chain

This finding was quite expected as soon as the intra-polymeric H-bonding could be depressed because of repulsion between negative charges of the anion-ionizable groups. Some relatively stable H-bonding was observed between C=O and H–O atoms of carboxylic groups predominantly in neighbor positions of polymeric chain (Scheme 3), where the H-bonds appeared within the flexible (succinic acid—SA) moieties more frequently and longer than at the rigid (cyclic—FU) fragments.

However, the main part of the ligand chain’s H-bonding capability was remained as unused resource (vacancies) accessible to intermolecular contacts. In theory, this peculiarity of the tested polymeric ligands could provide good preconditions for some additional H-bonds mediated interference with the viral target.

On the other hand, the target itself also has accessible “H-bond competent” vacancies (in side chains of amino acids). These vacancies are distributed among the three levels (L1, L2 and L3) of cavities/pockets as the theoretically allowed maxima of *D*_*Hb*_ = 38.8 (within L3), 32.5 (within L2), and 50.5 (within L1) (calculated from the data in Table 3). Based on this pre-calculation we could expect the following priority of the cavities levels to be attractive H-bonding niches for ligands: L1 > L3 > L2. In addition, an electrostatic attraction between the ligand’s polyanionic chain and target’s cationic side chains (Fig. 2) of lysine (on L1) and arginine (on L1 and L2) residues should be taken into account as well.

#### Ligand–target H-bonds formation, depending on target’s amino acids positions and nature

Above we demonstrated that the polypeptide backbone’s N and O atoms are generally involved in α-helix self-formation, and H-bond competent side groups of amino acids in ***a*** and ***d*** positions (of repeat heptad) contribute to the target self-assembly toward coiled-coil [HR1]_3_ complex. However, the target could be accessible for H-bond connection with any ligands, using other suitable vacancies at side chains of amino acid residues in ***b***, ***c***, ***e***, ***f***, and ***g*** positions of heptad repeat motifs, especially in respect of H-bond active side chains (Table 3).

This theoretical prediction is in good agreement with the MD generated experimental data: in Tables 5 and 6 one can see the dominant role of side groups of the ***b/c/e/f/g*** located Gln, Asn, Arg, His, Trp, and Lys in H-bonds formation with O atoms of the polycarboxylic ligands M_11_ and M_11+3dNb_. Simultaneously, no H-bond formation with these ligands was registered for side chains of any amino acids in the ***a/d*** positions of target.

**Table 5:**
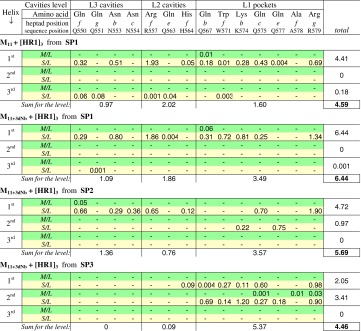
Degree of H-bonds formation between the target’s amino acids and the ligand’s species (O atoms of BU)

*Notes* The data are represented for the all MD-tested starting conditions (SP1–SP3) in dependence on nature of the involved target’s amino acid residues and their positioning in α-helixes (1st, 2nd or 3rd), HR1 polypeptide sequence (compare with Fig. 2), heptad repeat motifs (a–g) and cavities/pockets levels (L1–L3) in view of the target’s H-bond active centre location in the main polypeptide chain (**M/L**) or in side chains (**S/L**). By the M/L we designate the H-bonds between target’s main polypeptide chain (–**N**H–CR^1^R^2^–C**O**–) and O atoms of ligand, and S/L designates H-bonds of side chains of amino acid (the N/O atoms of R^1^/R^2^) with the same ligand. The “–” sign indicates that between the concrete amino acid and any ligand’s O/N atoms no H-bonds were recorded under the selected starting position (SP1/SP2/SP3)

**Table 6:** Target–ligand H-bonds degree distribution, depending on the target’s amino acid positioning in heptad repeat motifs of α-helixes

Ligand-Start\heptad position		*a*	*b*	*c*	*d*	*e*	*f*	*g*
M_11_	-SP1	0	0.98	0.43	0	0.004	2.41	0.77
M_11+3dNb_	-SP1	0	1.98	0.25	0	0.004	2.87	1.34
	-SP2	0	0.51	1.06	0	0.75	1.47	1.90
	-SP3	0	2.00	0.87	0	0.18	0.51	1.91
Total degree of H-bonds		0	5.47	2.61	0	0.94	7.24	5.92
Total probability (%)		0	24.7	11.7	0	4.3	32.7	26.7

#### Influence of ligand’s anchors on the ligand-target H-bonds capacity

In spite of the alicyclic dNb anchors didn’t possess any own H-bond competent atoms, notable indirect effects of these H-bond-inert species on the H-bonding were revealed. An influence of pendant anchors on the H-bonds formation between the target and ligand can be estimated by examples of the anchor-free M_11_ and anchor containing M_11+3dNb_ models in their comparative study at similar starting condition, the S1, for instance.

Unexpectedly, the MD modeling shown that the substitution of three “H-bond competent” OH carboxylic groups by the H-bond-inert dNb anchors in the [BU]_11_ polymeric chain resulted in no suppression of H-bonds formation. Quite the contrary, approximately 40 % growth of the ligand-target H-bonds intensity was observed (Table 5). The anchor containing M_11+3dNb_ (from SP1) provided *D*_*Hb*_ = 6.44 as compared with *D*_*Hb*_ = 4.59 induced by the M_11_ without any anchors. An epicenter of the H-bonds degree growth was located within the L1 niche of the target’s pockets, with detectable switching the H-bonds formation from L2 to L1 of the target’s cavities.

As the pendant anchors themselves can’t be direct partners in H-bonding, the observed fact may be explained by an enhanced tropism of the anchors to the hydrophobic pockets of L1. Covalently linked to polymeric chain, the anchors entrain the [BU]_11_ chain units toward this most attractive pockets, resulting in the anchors-mediated amplification of the polymer backbone H-bond contacts exactly in the L1 niche.

The similar synergetic effect of anchors was mentioned above in relation to the anchors-mediated intensification of all contacts between the ligand and the target (section “Role of the ligands’ anionic chain ([BU]_11_) and hydrophobic pendant anchors (Anc1–3)”). This very interesting discovery illustrates an opportunity of significant promotion of synthetic polymeric chains activity to bind biopolymeric (protein) targets due to pendant anchors, even if the anchors per se are not capable of H-bonding. Mechanisms and drug-design applicability of the polymer-cooperative specific activity will be discussed below (section “MD-based revision of the polymeric platform advantages for drug design”).

#### A centipede-like movement of poly(carboxylic acid)chain on the target surface

Possessing the oxygen-enriched chemical nature (44 carboxyl-derived –COX groups plus 11 ether –O– atoms, multi-repeated along the 11-meric chain of BU units), the relatively flexible polymeric backbone appeared very distinctive manner of interactions with the viral target in searching an geometry-energy optimal adaptation. This peculiarity can be defined as “*a centipede*-*like effect”* of the polymeric chain movement on the viral target’s surface due to the multiple H-bond active oxygen-based “legs”. And examples of such behavior were observed most evidently in dynamics of contacts of target’s side chains with sequences of oxygen atoms along the polymeric chain of ligands (via step-by-step analysis of sub-molecular contributions to the S/L H-bonding).

For instance, the side chain of high active Arg557 (within L2 of 1st helix) were involved in reversible stepwise H-bonding with the following sequences of O atoms of ligands models: O^37,38,39,40,41,42,43^ (M_11_ from SP1), O^27,28,29,30,31,32,33,-,-,36,37,38,39^ (M_11+3dNb_ from SP1), O^53,54,55,56,-,-,-,-,61,62,63,64,65,66,-,-,69^ (M_11+3dNb_ from SP2). The Lys574 (within L1 of 1st helix) side chain was stepwise filled by H-bonds with: O^61,-,63,64,65,66,67^ (M_11_ from SP1) and O^62,-,64,65,66,67,68,69^ (M_11+3dNb_ from SP1); the Arg579 (within L1 of 1st helix) side chain participated in H-bonding with ligand’s oxygen motifs: O^68,69,-,71,72,73,74^ (M_11_ from SP1), O^27,28,29,30,31,32,33,-,-,36,37,38,39^ (M_11+3dNb_ from SP1), O^53,54,55,56,-,-,-,-,61,62,63,64,65,66,…69^ (M_11+3dNb_ from SP2), etc.

These contacts (and snapshot series analysis) demonstrated reversible step-by-step translocations of the multiple oxygen “legs” (of the flexible ligand’s chain) through the H-bonds competent amino acids. The H-bond detectable points of amino acid residues, locally fixed on the target’s surface, allow of sensing this movement.

Comparing the behavior of M_11_ and M_11+3dNb_ models (from similar start, the SP1), we found that anchors rather assisted the main poly(acidic) backbone to be contactable with target. The anchors facilitated an involvement of more broaden diapasons of the oxygen (“legs”) motifs of synthetic polymer chain in this stepwise movement, amplifying an adaptability of the ligand to the target. This evidence represents another impressive example of mutual cooperation of BU and Anc units in interaction with the target (see below, section “MD-based revision of the polymeric platform advantages for drug design”).

#### Ligand-target H-bonds network from different starting positions

Broadening the field of view to full variations of the tested starts (S1–S3) (see Table 5), we should note that M_11+3dNb_ coiled SP2 conformation developed H-bonds network similarly to the SP1 start. The both SP resulted in axial orientation of H-bonds distribution—along the 1st helix predominantly. An additional and detectable contribution of contacts with 2nd helix (on the L1) in the case of SP2 was observed too. On the contrary, the SP3 starting conformation leaded to redistribution of the ligand-target H-bonds network from axial priority (along 1st helix within L1, L2 and L3) toward belting direction, involving both 1^st^ and 2nd α-helixes but accumulating the H-bonds within the narrow niche of pockets of the L1 mainly.

As it followed from the Table 5 data, a moderate total degree of the H-bonds (*D*_*Hb*_ = 4.59) between target and ligand was registered in the case of anchor-free ligand M_11_, start SP1. Similar start of anchor containing ligand M_11+3dNb_ resulted in the maximum H-bonding degree (*D*_*Hb*_ = 6.44) that was noted above in respect of synergetic intensification of H-bonding activity of the ligand carboxylic chain via the pendant anchors. Start SP2 from coiled conformation of M_11+3dNb_ leaded to a slightly reduced H-bonding degree (*D*_*Hb*_ = 5.66). And the same ligand transversally unfolded conformation from the start SP3 provided the lowest degree (*D*_*Hb*_ = 4.46), probably, because of the last start caused an occupancy of only one niche, the L1, without contributions of L2 and L3 H-bond capable vacancies.

#### Comparison of the target’s side-chains involvement in H-bond-mediated self-stabilization and binding with ligand

From the section "H-bonds of the target’s self-organization in the coiled-coil [HR1]_3_ complex" (Tables 2, 3) we can conclude that, having maximal potentiality to form H-bonds trough the [HR1]_3_ side chains (up to 141 H-bonds), the target used for coiled-coil self-stabilization only minor part of the degree *D*_*Hb*_ = 19.2 (i.e. 13.6 % of the full intra-target *D*_*Hb*_). Simultaneously, extracting the data from “Ligand–target H-bonds formation, depending on target’s amino acids positions and nature” to “Ligand-target H-bonds network from different starting positions” sections (Tables 5; 6), one can find that degree of H-bonds between the target and M_11+3dNb_ ligand is at even more reduced values of *D*_*Hb*_ = 4.5–6.4 (i.e. ≤4.5 %).

This preliminary analysis may lead to a tentative conclusion that ligand is able to use fewer H-bondable points of target than the target itself contributes into the own coiled-coil self-organization. However, it should be taken into consideration that above we dealt with statistic data accumulated during the full MD-simulated time (80 ns). The network of intra-target own H-bonds was completed as from first starting moment, while at the same starting time the modeled ligands had no H-bonds with the target. In contrast with the pre-filled intra-target network, the H-bonding network between ligand and target was being formed in processing during the all MD-simulated time, i.e. in the dynamic development from zero starting degree (*D*_*Hb*_^*start*^ = 0) toward a growing degree of H-bonds. Therefore, the statistics analysis should be completed by the dynamics evidences.

#### Some dynamics aspects of total H-bonds formation between the target and ligands

The MD simulated dynamics of H-bonds between the target and ligands, depending on the SP, is shown in Fig. 6 that is disposed in *supplementary material 2*.

Under the all discussed here MD-modeled systems the original amount (*Q*_*Hb*_^*start*^) and degree (*D*_*Hb*_^*start*^) of H-bonds between the target and ligands started from zero level. Along the simulated time these values was in progress, achieving by final 70–80 ns the following intervals of H-bonds instant quantity fluctuation (depending on starting conditions): *Q*_*Hb*_ → 4–7 and *D*_*Hb*_^*70*–*80* *ns*^ = 6 ± 1 (M_11_ from SP1); *Q*_*Hb*_ → 8–12 and *D*_*Hb*_^*70*–*80* *ns*^ = 9 ± 2 (M_11+3dNb_ from SP1); *Q*_*Hb*_ → 7–11 and *D*_*Hb*_^*70*–*80* *ns*^ = 9 ± 2 (M_11+3dNb_ from SP2), and *Q*_*Hb*_ → 4–8 and *D*_*Hb*_^*70*–*80* *ns*^ = 7 ± 2 (M_11+3dNb_ from SP3).

The anchor-containing ligand M_11+3dNb_ manifested the higher potency in comparison with the anchor-free precursor M_11_ (Fig. 6 in *supplementary material 2*). Among the MD-tried starts of the M_11+3dNb_ the best H-bonding was registered from the SP1. The SP2 provided less dynamically active H-bonding, and SP3 demonstrated rather not very fast (but most uneven) temp of H-bonds formation with moderate level (like to the M_11_ from the SP1 start).

Generally, the discussed order of H-bonds dynamics is in good agreement with mentioned statistics data. However, taken into account the dynamic-probable prognosis to further growth of the H-bonding beyond the applied limit of MD-simulated time (80 ns), we can state a very considerable potency of the ligands to develop H-bonds network with the viral target. Therefore, from the dynamics point of view, the statistics-based pre-conclusion can be corrected toward more multiple (and therefore, the more potent) H-bonding between ligand and target. It is right especially for the H-bonds development by anchor-equipped M_11+3dNb_ up to levels comparable with the H-bonds degree used by the target for the coiled-coil self-assembly.

### Dynamics and statistic of energetic contributions in the ligand–target binding

Besides the above considered statistics and dynamics data, even more interesting aspect (undetectable via docking) is evolution of energies of the various contacts in dynamics. The ligands–target binding energy nature and a comparative role of Anc and BU components in cooperative interaction with target become more evident from analysis of their contributions to the energy of the target binding. Analyzing this aspect, we focused on the M_11+3dNb_, as model of polymers **I** sub-type most relevant in the HIV-1 inhibition.

#### Partial contributions of various kinds of energy to total binding energy

The MD-simulated development of ΔG_Bind_ and their filling by the partial contributions of van der Waals (**E**_**vdW**_), Coulomb (**E**_**Q**_), and solvation (**G**_**Sol**_ = **G**_**polar**_ + **G**_**nonpolar**_) forces is demonstrated in Fig. 7 disposed in *supplementary material 3*.

The most significant minimization of the ΔG_Bind_ was promoted via the Coulomb forces E_Q_. This result is in good agreement with the chemical nature of the modeled objects as the mutually-attracted carriers of opposite charges. The target represents the cationic domains [HR1]_3_ of gp41 biopolymers, while the ligand’s backbone is unlikely charged (anionic) polycarboxylic acid.

The Coulomb forces could be dominant part of the ΔG_Bind_ if the interactions between the target and ligand occurred in vacuum. However, for modeling a physiological condition, a polar solvent (water with a presence of Na^+^, see experimental part) should be taken into account too. The electrostatic-relevant polar component G_polar_ of solvation energy contributed to enhancing the ΔG_Bind_, i.e., toward dissociation—against the Coulomb binding. And summation of E_Q_ + G_polar_ resulted in moderate increasing the ΔG_Bind_ (up to 50 kcal/mol) more preferable for unbinding. Therefore, the MD-based estimation of the Coulomb/electrostatic contributions only leaded to an expectance of rather dissociation than binding between the modeled target and ligand.

The nonpolar part of solvation energy G_nonpolar_ provided the slightest effect on the level of ≤ |-10| kcal/mol, modulating the ΔG_Bind_ to a very little degree.

The main resulting contribution to ΔG_Bind_ was filled by the van der Waals forces. The E_vdW_ minimizing to interval from −50 to −80 kcal/mol was achieved by an orientation of ligand contacts along the L1–L2–L3 zones of α-helix (SP1 and SP2 starts). The minimal E_vdW_ (−100 kcal/mol) was observed from the SP3 start, which directed interactions of ligand toward L1 pockets (the largest-deepest cavities of the target). But this contribution of E_vdW_ for binding under the SP3 conditions was compensated by the high counter-contribution of E_Q_ + G_polar_ to dissociation at the same start. It resulted in comparable values of the summarized ΔG_Bind_ for the all tested starts of M_11+3dNb_. In the long term, the resultant ΔG_Bind_ evolved as a function of starting conditions from zero level to the following values (kcal/mol, statistically averaged for 70–80 ns interval): −63.0 ± 9.7 (SP1), −59.1 ± 12.5 (SP2) and −61.8 ± 14.5 (SP3).

However, such, in principle useful, consideration given too generalized information without any special analysis of genesis and balance of these energies contributed by different components of the ligand molecular structure, notably, the anionic backbone and the pendant anchors.

#### Energetic contributions of polymeric chain ([BU]_11_) and anchors (Anc1–3)

Before the declared analysis we should note some differences between the polymeric chain backbone and the pendant anchors in relation to their chemical nature and potentiality: (1) the chain is electrostatic (anionic) active part, while the anchors possess no charged atoms in practice; (2) the polymeric chain is the major part of the ligand molecule, containing fourfold more amount of atoms than the three anchors taken together; (3) the backbone’s units, back-to-back linked into linear polymeric chain, are very limited to be contactable with target independently on the chain sequence and configuration, while the every single anchor is linked to the backbone through flexible bridge (–NH–CH_2_–), possessing more freedom for a mobility in the contacts. Based on these peculiarities of molecular organization we could expect: (1) dominant contribution to Coulomb and polar solvation interactions by the [BU]_11_ component, but not by the anchors; (2) a contribution to van der Waals interactions by the [BU]_11_ approximately 4 time higher than one by the anchors; (3) more evident dynamic mobility of anchors in contacts with target, as compare with mobility of polymeric chain.

All these prognoses met with experimental confirmations by the MD-simulated results (Fig. 7 - in *supplementary material 3*, and Table 7).

**Table 7:** Energetic contributions (kcal/mol) of polymeric chain ([BU]_11_) and anchors (Anc1–3) to binding (negative values) or dissociation (positive values) in interactions between M_11_/M_11+3dNb_ and [HR1]_3_

Ligand	Ligand’s component	Start	ΔG_Bind_=	E_vdW_	+G_nonpolar_	+G_polar_	+E_Q_
**M**_**11**_	[BU]_11_	SP1	↓−29.4 ± 9.2	↓−44.2 ± 4.8	↓−5.3 ± 0.3	↑+585.0 ± 119	↓−565.0 ± 122
M_11+3dNb_			↓−56.8 ± 5.9	↓−38.4 ± 5.0	↓−5.6 ± 0.4	↑+958.6 ± 130	↓−971.4 ± 131
		SP2	↓−41.7 ± 7.3	↓−47.2 ± 6.1	↓−6.2 ± 0.5	↑+1,098.8 ± 133	↓−1,087.0 ± 134
		SP3	↓−46.5 ± 8.1	↓−60.2 ± 5.5	↓−6.7 ± 0.4	↑+631.3 ± 115	↓−610.9 ± 119
**M**_**11**_	No Anc	SP1	0	0	0	0	0
M_11+3dNb_	3 Anc		↓−6.2 ± 2.1	↓−17.4 ± 2.8	↓−1.2 ± 0.2	↑+185.5 ± 27.1	↑−173.1 ± 27.6
		SP2	↓−17.5 ± 2.8	↓−22.7 ± 2.5	↓−1.7 ± 0.1	↑+11.5 ± 7.8	↑−4.6 ± 7.9
		SP3	↓−15.3 ± 3.0	↓−30.5 ± 2.7	↓−2.1 ± 0.1	↑+198.3 ± 25.6	↑−181.0 ± 25.4
**M**_**11**_	Complete molecule	SP1	↓−29.4 ± 9.2	↓−44.2 ± 4.8	↓−5.3 ± 0.3	↑+585.0 ± 119	↓−565.0 ± 122
M_11+3dNb_			↓−63.0 ± 9.7	↓−55.8 ± 5.3	↓−6.8 ± 0.3	↑+1,144.0 ± 131	↓−1,144.4 ± 134
		SP2	↓−59.1 ± 12.5	↓−69.9 ± 6.7	↓−7.9 ± 0.5	↑+1,110.2 ± 133	↓−1,091.6 ± 135
		SP3	↓−61.8 ± 14.5	↓−90.7 ± 6.8	↓−8.8 ± 0.4	↑+829.6 ± 118	↓−792.0 ± 122

*Notes* The markers ↓ or ↑ indicate tendencies to alter energy from starting zero level toward decrease (factor for binding) or growth of energy (factor for dissociation), respectively; and on the right the respective negative or positive amplitudes of energy values (kcal/mol, averaged for the 70–80 ns intervals of the MD simulated time) are represented

##### Contributions to E_Q_ and G_polar_

The strong and dominant role of anionic backbone in Coulomb interactions (as well as in polar part of solvation energy) appeared evidently: the averaged (within 70–80 ns) contributions with amplitude up to 1,144 kcal/mol by the [BU]_11_ against ≤200 kcal/mol by the anchors (Table 7).

##### Contributions to E_vdW_

Major part of the van der Waals forces energy was generated also due to the [BU]_11_ contribution (38–60 kcal/mol, averaged amplitude for 70–80 ns). But the anchors contributed more considerable (17–31 kcal/mol) than it could be expected from the 1:4 proportion of atoms amount in the Anc1–3 and the [BU]_11_ components, respectively. The observed proportion ~1:2 (enriched by the anchors’ contribution) can be explained exactly through the above assumed enhanced contactable mobility of pendant anchors in contrast with the [BU]_11_ chain. If the chain backbone is the less mobile construction it has require more long time for adaptation on the target toward the binding energy sub-minimum than the time of anchors’ adaptation. In Fig. 7 (*supplementary material 3*) we can see that is really so.

##### A comparison of M_11_ and M_11+3dNb_ in interactions with the same target at the same start (S1)

It given the valuable information: how a presence/absence of the pendant anchors did alter the energetic contributions to binding the target? This comparison of two different ligand molecules was a light additional to the estimation of comparable contributions of anchors and [BU]_11_ within one molecule, the M_11+3dNb_ only. It was revealed that the three anchors (in M_11+3dNb_ against anchor-free M_11_) altered amplitudes of energies considerably, as the following (Table 7): 203 % (−E_Q_), 196 % (G_polar_), 128 % (−G_nonpolar_), 126 % (−E_vdW_) and 214 % (−ΔG_bind_).

In contrast with the noted intramolecular (M_11+3dNb_) proportion of partial contributions to E_vdW_ by the anchors and [BU]_11_, as 1:2, a comparison of the two different molecules (M_11+3dNb_ and M_11_) indicated the decreased proportion (Table 7): (55.8–44.2) : 44.2 = ~1:4. The last proportion was exactly equal to the expected one in relation to ratio of atoms in anchors and atoms in [BU]_11_ chain. Therefore, the addition of anchors’ atoms (from M_11_ toward M_11+3dNb_ molecules) increased the ligand ability to interact with target via van der Waals forces proportionally to the growth of atoms amount. But resulted entire molecule M_11+3dNb_ realized these interactions via the “leader-enhanced” contribution of anchors. This result agrees also with the conclusion about more active mobility of just anchors in contacts with the target.

Moreover, it was interesting to find that the nonpolar anchors twice as much intensified both Coulomb energy and electrostatic term of solvation energy. This finding is another, very evident, confirmation of highly active role of the pendant anchors in interactions with the target. The involvement of anchors stabilize simultaneously a connection of poly(acid) chain with the same target. And this anchor-induced stabilization results in significant minimizing the −E_Q_. Apparently, being independent on electrostatic-inert anchors directly, the polar interaction depends on intensity of [BU]_11_—target contacts, which are accelerated and amplified by the anchors, see sections “Role of the ligands’ anionic chain ([BU]_11_) and hydrophobic pendant anchors (Anc1–3)” and “Influence of ligand’s anchors on the ligand-target H-bonds capacity”.

##### Dynamic differences in the energies filling by the [BU]_11_ and Anc1–3 contributions

As it followed from the data in Fig. 7 (*supplementary material 3*), the Anc(s) linked through the flexible bridges to backbone were distinctly more mobile (than BU) agents for initial contacts with the targets. Their contributions to −E_vdW_ energy of binding with the target achieved the minimum during very short time (*T*_*min*_^*A*^ ≤ 5–10 ns), while the same for the [–BU–]_11_ required longer period of time (*T*_*min*_^*B*^ ≥ 20 ns) (Fig. 5). Like the E_vdW_, the full ΔG_Bind_ reproduced this difference: the partial contribution of anchors was filled more rapidly than the chain backbone contribution. Analogically, within the MD snapshots-based conformations analysis the anchors were ahead of time in contacts with target, while the backbone was late (for example, Fig. 4). Moreover, the dynamic behavior of the [BU]_11_ in process of both H-bonds formation and the partial energies filling, allowed to suppose that these processes didn’t achieve a completeness by the final time point of the MD-simulation (80 ns), keeping a potentiality for probable growth beyond the simulated time.

##### Comparison of the binding energy, estimated via the MD and docking

Both MD- and docking-based estimations of binding energy between the anchors containing anionic polymers (modeled via M_11+3dNb_) and the viral target [HR1]_3_ resulted in values adequate to provide a powerful binding the viral target at the physiological temperatures. But the docking-estimated binding (Fig. 3) were stronger than MD-simulated ones. It was relevant result so long as the docking procedure modeled the energetically minimized conformations, while the MD simulated a dynamically developing process toward these minimums. Additionally, the MD taken into account destabilizing effects of Brownian motion as well. But the used time of MD simulation could be too short to achieve the best (with minimal −ΔG_Bind_) conformations predicted via docking. It can be noted that the simulated time, the 80 ns, is approximately 10^10^-folds shorter than a full time required for HIV-1 virions to complete the fusion step of entry into cells, estimated within about 15 min [42]. MD simulation of so long time needs too prolonged computational time quite beyond the currently available resources of MD.

However, even the used 80 ns simulation by MD resulted in sufficiently great values of −ΔG_Bind_. And the conformations generated via MD confirmed generally the main binding sites and modes, predicted via the docking.

### MD-based revision of the polymeric platform advantages for drug design

A great role of polymeric compounds as a basis for traditional small molecule drugs improvement through polymer-coupled drug-delivery/release strategies is well-known [43]. Keeping this aspect without any additional consideration, in conclusion of the current paper we would like focus on fundamental differences between small and polymeric molecules in own potentialities for a drug design. This problem is most needed in view for feasible advancement from limitations toward development of therapeutic effectiveness, and from predisposition toward prevention of a drug resistance.

#### Size adequacy of therapeutic ligand to the biological targets (the nano-competent drugs)

One of the fundamental limitations of small molecules effectiveness, as target-blocking therapeutic agents, is the size inadequacy between such molecules (small ligands) and the targets. A majority of the biomedical relevant targets represent biopolymeric macromolecules (proteins, nucleic acids, etc.) self-assembled in nano-complexes. For example, the considered in this work protein-type viral target [HR1]_3_ is a typical nano-scale (2.5 × 5.1 nm) object.

Apparently, no any small molecule can be fully effective blocker of same target, in principle, because the small ligand can connect with only small part of this target, but can’t cover main/full binding vacancies on the surface of macromolecular target. Therefore, the size inadequacy becomes an objective barrier of the modern drugs efficiency development, if the drugs are small molecules designed specifically for the blockage-of-target therapy.

Exactly this is the cause of slight or only moderate efficiency of great number of small molecules screened for the HIV-1 entry inhibition through mechanisms of binding the gp41-related mediators of fusion. No significant anti-HIV effects were found in our previous investigations [3–7] and other researchers works [44, 45] among small molecule-type precursors, or analogous of the modeled polymers of formula **I**. Particularly, the small molecules, chemically related to the cage alicyclic pendant anchors, were anti-HIV ineffective [3–7, 45]. Although many among such compounds (*amantadine, rimantadine, deitiforinum* etc.) are well-known inhibitors of influenza type A viruses, this activity we interpret as a result of these small ligands penetration into the viral proteins M2 complex (the intra-target intervention) [47], but not due to any external binding the target, that is impossible in fact of the discussed here nano-scale inadequacy.

In contrast with the small molecules, the pendant anchors containing polymers of formula **I** (Scheme 1) are high selective and efficient inhibitors of the HIV-1 entry in vitro [3–7]. *In silico* modeling via our suggested step-by-step docking algorithm [9, 16] for interactions between synthetic and biological polymers (by example of the polymers **I** and the viral [HR1]_3_ target) explains the in vitro recorded data with good correlation. The docking pre-study led us to clear understanding the role of the ligand–target size adequacy in concrete terms of geometrical parameters, required to strong binding this target axially or by belting via the polymeric compounds in contrast with small molecules, modeled as fragments of these compounds (Fig. 3).

In this paper the docking predicted size of the polymeric ligands needed for axial (≥5.1 nm) and belting (≥8 nm) binding the [HR1]_3_ (see in Fig. 3), was verified in the MD procedure by examples of the 11-meric chain-based models of polymeric ligands (the M_11_ and M_11+3dNb_). The MD simulation, generally confirming the docking prediction, demonstrated additionally a dynamic evolution of the forecasted intervention of synthetic polymers **I** in interactions with the viral target. This intervention was modeled from various SP and considered in relation with synergic role of mutual cooperation of anionic polymeric chain and pendant anchors, where the both BU monomers and individual anchors represented, separately, the small molecules themselves ineffective against HIV.

Therefore, the following postulate can be accepted: the geometric scale of a ligand (drug) comparable with the molecular scale of a target is the precondition crucially needed for a maximally realizable efficiency of binding this target (through an optimization of the ligand’s chemical structure within this size adequacy).

#### From separate small molecules toward synergic-cooperated polyligands (the multipoint-binding drugs)

So the small molecules are very limited sours for a drug design of agents for blocking the (bio)macromolecules ((bio)nano-objects), in principle. On the other hand, as we asserted before [8, 47], the same small molecules can be mostly useful agents as antimetabolites (in competition with natural low-molecular mass metabolites) or as antagonists/inhibitors of active centers of bio-receptors or enzymes, if these centers are precisely adequate to the small molecules geometry. On the contrary, a full inhibition of such centers simultaneously with relevant allosteric co-factors of entire macromolecule (or macromolecular complex) needs just the size-comparable polymeric agents preferable. And exactly the macromolecular poly-ligands can be the best therapeutic tools to cover main surface of target, if the target should be efficiently blocked by means of binding. In this relation, the macromolecular approach to drug design is a strategic priority of our research group [7–10, 47].

Below we would like demonstrate an appropriateness of this proposition by detailed examples from the docking and MD co-investigation of the considered molecular objects.

##### Mutual cooperation of the modeled ligand’s components in binding the target

As followed from the docking pre-study [9, 16, 48] (Fig. 2), the small-size precursors of polymers **I**,^10^ modeled as a single small molecule, were capable of cowering simultaneously only a narrow local part of full contactable surface of the viral target (Fig. 2, step 1). And the best binding energy (simulated via docking) was observed in contacts within one from deepest pockets at the 1st level (L1) pockets triplet of the target. However, any from among such contacts between the “small ligand” and the “big target” achieved binding energy no more than |−ΔG_Bind_| ≤ 20 kcal/mol (ibid). This is too slight energy to support a stable binding under physiological temperatures (because of Brownian movement). Thus, the computer-aided modeling cleared the cause of the mentioned experimental facts of the anti-HIV inefficiency of the real small molecules, evaluated in vitro [3–10].

A subsequent modeling the (olygo/poly)meric ligands, using both docking (Fig. 2, steps 2 and 3) and MD techniques, evidently revealed a significant growth of achievable values of |−ΔG_Bind_| > 50 kcal/mol (Fig. 3, Fig. 7 in *supplementary material 3*, and Table 7). This is quite enough energy to provide the very stable binding (and the anti-HIV protection in vitro observed). Certainly, the binding power growth should be logical result from the size escalation of a ligand molecule (especially due to the E_vdW_ contribution), but if the ligand’s sub-structural units are in cooperative synergism (or additivity), and not in crucial antagonism (competition) within interactions between the ligand and target.

The main principles for a synergistic macromolecular drug design were formulated and studied, particularly, in our previous works [7–9, 16, 47–50], and now they are cleared and developed on the platform of the current computer-aided modeling.

As one could see above, the antiviral active polymeric ligands of series **I** were designed as a cooperation of many small anionic units BU toward linear and flexible enough chains [BU]_*n*_ followed by grafting the special pendant anchors at certain distances along these chains.

*The poly(carboxylic acid) chain* nature was chosen in view of nature of the viral protein target: (1) to be electrostatic-attractive to the target through interactions between the negatively ionizable carboxylic groups and counter-ionizable groups of side chains of Arg/Lys/His residues of target, as well as (2) to be able to form multiple H-bonds with the target due to multi-repeated along the ligand’s chain H-bond-active oxygen atoms (Table 4, for instance). The 11-meric length of the polymeric chain for the MD testing was selected on the base of the docking prediction (Fig. 3): (1) to be able to cover more than full length (>5.2 nm) of a single α-helix of [HR1]_3_, using all three levels of the target’s cavities/pockets (the L1, L2 and L3), as well as (2) to be capable of belting the target.

*The pendant alicyclic anchors* chemical nature was originally (at step of design for synthesis) selected: (1) in contrast with nature of the polyanionic chain to be not rival (in respect to electrostatic or hydrogen bonds), but to be complementing component oriented toward hydrophobic sites of targets; and (2) in view of a background of adamantane and norbornane-related alicycles as synthetic core for antiviral compounds (well-known mostly in anti-influenza therapy [52], but not in anti-HIV treatment). Among the possible alicyclic structures the dNb species and their dislocation in side positions of polyanionic chain was selected by in vitro screening [3–10] in search for most anti-HIV-1 active synthetic polymers of the series **I** followed by the docking-based analysis [9, 16] (Fig. 3, for example).

Resuming the discussed in this article MD simulation, we accumulate the following relevant manifestations of the ligand’s components synergetic cooperation in relation to an amplification of the binding with target.

1. The cooperation of monomer units BUs toward 11-meric chain (the [BU]_11_) leaded to:
  11-folds multiplication of the ligand capacity of multi-point binding with target;
  expanse of geometrical ability to cower simultaneously not single cavity/pocket but all three levels of cavities/pockets along full length of the target’s α-helix, or to belt the target;
  centipede-like movement of poly(carboxylic acid)chain on the target surface (section “A centipede-like movement of poly(carboxylic acid)chain on the target surface”);
  growth of amplitude of energetic contribution to total binding energy up to |−ΔG_Bind_| = 50–75 kcal/mol, and, probably, more (section “Energetic contributions of polymeric chain ([BU]_11_) and anchors (Anc1–3)”)
2. The grafting of three pendant anchors of dNb-type to the [BU]_11_ chain resulted in:
  twice more intensive contacts between ligand and target by virtue of the anchors-induced 14 % growth of amount of BUs-target contacts plus 93 % increase due to additional anchors (themselves)-target contacts (section “Role of the ligands’ anionic chain ([BU]_11_) and hydrophobic pendant anchors (Anc1–3)”);
  half as more intensive H-bonding (section “Influence of ligand’s anchors on the ligand-target H-bonds capacity”);
  assistance for [BU]_11_ to be more contactable with target via facilitating an involvement in centipede-like movement on the target’s surface (section “Role of the ligands’ anionic chain ([BU]_11_) and hydrophobic pendant anchors (Anc1-3)”);
  a considerable additional contribution of anchors into binding energy, especially, through the van der Waals forces (section “Energetic contributions of polymeric chain ([BU]_11_) and anchors (Anc1–3)”); and, at last,
  the accelerating effect of anchors on BU species involvement into synergetic contacts with target in dynamics.

Really, the presence of anchors provided very relevant (but undetectable via docking) effect on BU species involvement into synergetic contacts with target in dynamics. The anchors demonstrated a leading mobility as a time advanced agents for initial binding with target, followed by the next anchor-induced contacts with target of chain units linked to anchors (by bridges). Then other BU step-by-step are involved (through the back-to-back co-linkage of the BU monomers into the polymeric chain). The capability of anchors to be quickly contacting initiators in interactions with target was detected in focus on both mechanical and energetic manifestations (sections “Dynamic evolution of distances between ligand’s and target’s substructures” and “Energetic contributions of polymeric chain ([BU]_11_) and anchors (Anc1–3)”, respectively).

In fact, via the MD simulation, we revealed the essential role of anchors as factors of acceleration and amplification of the binding between the polymeric molecules and the viral target. This finding opens an evident explanation of the early reported [3–10] experimental fact of enhanced anti-HIV activity in vitro of the anchor containing polymers in comparison with anchor-free precursors (or small molecules related to single anchor or BU).

Thus, the Anc(s), being less energetically powerful than full polymeric backbone (section “From separate small molecules toward synergic-cooperated polyligands (the multipoint-binding drugs)”), nevertheless, play very important role initiating first contacts with the target, and promoting subsequent involvement of BU(s) in the same interactions.

On the other part, multiple BU(s) stabilization on the target surface leaded to more stable anchors—target contacts too. As a result, even those local contacts, which were estimated as week—unstable (in the docking of small molecule models of single BU/Anc species) [9, 16], in the MD were re-qualified as quite stable [18] in virtue of the polymeric organization, that cooperate such species together. A local lost of contacts of any single Anc/BU with the target were reversibly restored in dynamics due to other (neighbouring) BU(s)/Anc(s) connections with the same target.

This situation can be illustrated in visible details via step-by-step analysis of the MD simulated snapshots in dynamics (of 0.1 ns intervals). As soon as the full sets of 800 snapshots (for every start) are too extensive data base, we represent some brief description only (see *supplementary material 4*). It demonstrates evidently that the polymeric coupling of small molecule units leads to a cooperation which is very relevant for behavior and functionality of the units in the “polymeric team”. Under the cooperation both antagonistic and additive/synergic effects in local and summarized interactions of components of polymeric ligands with targets can be fulfilled, depending on molecular design. Basing on the considered results of MD analysis we should state that the selected molecular structure of M_11+3dNb_ is enough optimal design to promote rather the synergism than antagonism in binding the tested viral target.

In general case, if the molecular architecture is successful, the cooperative potency of polymerized molecular systems becomes a crucial fundamental advantage of polymeric compounds in comparison with small molecules. And, therefore, this fundamentals cannot be excludes from theory and practice of novel materials development, including the drug design.

#### Drug efficiency and drug resistance

The cooperatively accelerated and amplified ability of M_11+3dNb_ to bind the HIV-1 fusion mediator [HR1]_3_ (in the represented MD simulation) supplies clear explanation of high efficiency of compounds **I** (Scheme 1 where X = active anchors in amount of 6–8 % among all X) as inhibitors of the virus entry into cells, and, therefore, as a drug-capable preventive agents for anti-HIV/AIDS therapy.

But the *preventive/therapeutic efficiency* is only one side of a global problem in area of drug design. The other side of the matter (that becomes more and more dangerous barrier for antimicrobial drugs development) is a *drug resistance*. No any drug design based on small molecules platform, exclusively, can ensure a cardinal solution of this problem.

Why is it? It is so because even *a combination of small molecules is not their “covalently*-*coherent cooperation” in contacts with a macromolecular target.* The covalently unbound (non-cooperated) small particles act independently (at absence of any coordinating forces). And Brownian movement, chaotically dispersing them, does not allow a combination of small molecules to act *simultaneously* against a target. But if the small species are covalently pre-cooperated together in some polymeric molecule they become capable of mutually-synchronized intervention in contact with other macromolecule (a target).^11^ Without this covalent co-linkage (“the polymer-specifically determined cooperation”) any abilities of small molecule drugs to full inhibition of such targets are strongly limited by the size-inadequacy of single small molecule (section “Size adequacy of therapeutic ligand to the biological targets (the nano-competent drugs)”) and by non-coordination of any different small molecule drugs mechanically combined for a therapy.

That’s why even modern combined antiretroviral therapy (included in HAART strategy through anti-HIV/AIDS “cocktails”) is not fully effective and very liable to drug resistance, provoking more and more dramatic multi-drug resistance in long-term therapy [1, 53].

In spite of a drug *resistance itself is caused by mutations* (of viruses, for instance), *the yield of any drug resistant mutants is crucially limited by efficiency of the drug* to block reproduction of the mutable microorganisms (viruses). A probability of the drug resistant mutant genesis depends of scale of part of the target that is blocked by the drug. If some small molecule drug controls only adequately small part of polypeptide sequence of target, just the one (point) mutation can be enough to generate resistance against such drug. And probability of the single mutation is rather high.

In contrast with small molecules, antibodies (the natural polymeric ligands for selective binding) interfere with more long sequences (antigen determinants) of targets. Quite more large part (or even full macromolecule) of target can be covered by binding through the cooperative manner, realizable via specially designed synthetic polymers, similar to the polymers of series **I** [8]. To become free from drug control of such polymeric inhibitors, not one-point but *many*-*points mutations are needed simultaneously*. A probability of the drug resistance against multipoint-binding drug *a priory* should be many folds less, as the product of probabilities of point mutations along all sequence of target.^12^

In focus on the tried target [HR1]_3_, the mediator of HIV-1 entry (fusion step), we should accentuate the following findings: (1) any small molecule ligand is able to bind adequately small site of the target, only at either L1, or L2, or L3, but not two or several sites simultaneously (pre-studied via docking); (2) polymeric ligands based on chain backbone, length of which is comparable with (or more than) nano-dimensions of the target, are capable of covering full-length α-helixes, the L1, L2 and L3 (36 amino acids sequence) simultaneously due to the discussed here cooperation of BU species and pendant anchors.

This cooperation for multipoint binding the viral target by the polymeric (type **I**) ligands is detectable in space (in the geometric and energy terms) via both docking and MD modeling, while the same cooperation in-time (in dynamic evolution of reversible contacts toward binding network) is undetectable via docking. It becomes clearly visible in MD. Therefore, taken together the docking and MD data, the polymeric type **I** inhibitors of HIV infection can be expected to be capable of a significant suppression of yield of drug resistant viral mutants. In theory, this is estimated effect of the cooperative (multilevel) blocking the more extended areas (and mutagenesis risks) of viral target(s).

In fact, this finding (pre-formulated previously [7, 8], tried by the docking [9, 16] and verified via the MD) is in a good agreement with the experimental result of in vitro evaluation of the polymeric sample of series **I** (where Anc = Ad), closely-related to the M_11+3dNb_. No significant resistance of HIV-1 mutants to this anti-HIV-1 active compound was achieved during long-term (40 days) experiment [7].

## Conclusions

1. Complementary co-application of the docking and MD is the very productive and improvable approach to in-depth modeling and investigation of interactions between synthetic and biological polymers. The step-by-step algorithm of docking [16] allows to find mainly probable sites and modes of binding the bio-polymeric target by synthetic polymer ligands and to estimate the binding energy minimums. Besides, it gives useful orientation for planning of following MD experiment (options for starting conformations, e.g.). The docking-based results and extrapolations can be verified and developed appreciably by the MD via simulation of larger scale molecules of polymeric ligands, taking into account a conformational flexibility of both synthetic (“ligand”) and biologic (“target”) polymers, as well as evolution of their interactions in time.
2. By the example of HIV-1 envelope glycoprotein gp41 nano-complex of [HR1]_3_, the virus fusion mediator, as a target, and 11-meric polyelectrolyte chain contained three pendant anchors of dinorbornene (M_11+3dNb_, the representative model of highly active inhibitor of HIV-1 entry), and its anchor-free precursor (M_11_, the model of weakly active anti-HIV inhibitor), the both as ligands, the following findings were revealed.
  2.1 The target in presence of the tested ligands under physiologically relevant temperature maintained generally own self-organization as the coiled-coil three α-helixes nano-complex (5.1 × 2.5 nm). In Brownian fluctuations it kept the classical order of self-formation (due to H-bonds between *i* + 4 NH and *i* O=C pairs of polypeptide backbone) of α-helixes, which then self-assembled in the three-helix coiled-coil complex [HR1]_3_, using the side chains of amino acid residues in ***a*** and ***d*** positions of heptad repeat motifs.
  2.2 The amino acid residues in ***b***, ***c***, ***e***, ***f***, and ***g*** positions of heptad repeat motifs (especially in respect of H-bond and/or hydrophobic active side chains), unused in the intra-target self-organization, were found to be accessible and active points for binding with the external polymeric ligands.
  2.3 Clarifying the target’s binding capability, the MD verified (from variable starting conformations) the pre-identified via docking main sites and modes of a probable binding between the target and the ligands. In fact, the MD confirmed both the general binding locus at first level (L1) of target’s pockets and additional sites at second (L2) and third (L3) levels of cavities that together provided a network of active vacancies for an occupation by ligands. The MD corroborate also the docking-predicted probability of the ligands attachment by the modes for axial covering (the all levels L1–L2–L3 along α-helix of target) or for belting around the level(s).
  2.4 Verifying the docking (step 3) extrapolation toward ligands with enough long-length polymeric chains, the MD demonstrated the ***principle of size*** **-** ***adequacy*** between target and ligand to provide a full-scale occupation-binding of the target. Particularly, the docking-predicted ability to cover both lengthwise and belting dimensions of the nano-target by the 11-meric chains of M_11_/M_11+3dNb_ models was observed in MD simulation. Just the polymeric chain size expands a geometrical ability to cover simultaneously not single site/cavity/pocket but all three levels of cavities/pockets along full length of the target or to belt the target. Taken together, the docking and MD results demonstrate the ***principle of size*** **-** ***adequacy*** as a fundamental advantage of polymeric compound (against small molecules) to be essentially more efficient agents (ligands) in drug design for a binding-type arrest of biopolymer (nano)targets.
  2.5 The *diversity, multiplicity and synergism* of contacts with a target, which can be realized through ***polymeric*** **-** ***type cooperation*** 13 is another fundamental advantage of the polymeric ligands for a drug design. In this relation the MD generated new data in addition to the docking pre-studied effects of possible mutual synergism/additivity/antagonism between various sub-structural units (components) of a ligand in binding with target, depending on the ligands’ molecule design. The MD simulation of the most anti-HIV active polymeric sample by the M_11+3dNb_ model, for this concrete case, demonstrated evidently an expressive trend to priority of the target-binding synergism between their sub-molecular components: the anionic monomer units (BU) co-linked in the quite flexible polymeric chain (the backbone) and pendant anchors grafted by bridges to this chain at the optimal distances.
    The polymer-cooperated accumulation of the target-binding potential was registered through at least following manifestations: (1) 11 folds multiplication of the ligand’s capacity of many-point binding with target due to the 11-meric repeating the BU units along the polymeric chain itself; (2) ability of the poly(carboxylic acid)chain to move on the target surface via centipede-like manner; (3) crucial role of the pendant anchors as additive and/or synergetic factor for acceleration, amplification and stabilization of multipoint binding the target. The only three anchors (grafted to M_11+3dNb_ in contrast with M_11_) resulted in twice more intensive contacts between ligand and target, half as greater H-bonding, and considerable additional contribution into binding energy. Moreover, the significant accelerating effect of the anchors on involvement of BU species into contacts with target and the mutual adaptation on the target surface in dynamics were observed.

The all noted findings, taken together, give new information very important for elucidating the molecular mechanisms of the modeled inhibition of HIV-1 entry by the real synthetic polymers [3–10], explaining causes of their enhanced antiviral efficiency and drug resistance prevention as opposed to small molecule analogues. Therefore, the obtained knowledge is a valuable platform for the novel drug design based on synthetic polymeric compounds in view of discussed here fundamental advantages of polymers in contrast with (and in addition to) “traditional” small molecule drugs.

Some unconsidered aspects of a computer-aided modeling, synthesis and bio-evaluations of same and other polymeric compounds-ligands (with variations of polymeric chains structure/flexibility and nature/functionality of side anchors/branches, etc.) will be represented in our subsequent publications.

## Electronic supplementary material

Below is the link to the electronic supplementary material.

Supplementary material 1 (DOC 60 kb)

Supplementary material 2 (DOC 127 kb)

Supplementary material 3 (DOC 600 kb)

Supplementary material 4 (DOC 51 kb)

## Footnotes

1 AIDS—acquired immunodeficiency syndrome, the disease caused by the HIV infection.

2 on experimental models of various HIV-1 strains infecting cells.

3 The binding selectively driven by counter-ionisable (cationic) Lys574/Arg579 at L1, His564 at L2, and Arg567 at L3, respectively.

4 Using the recently suggested algorithm, adapted for the polymer–biopolymer interactions modelling [16].

5 The required simulation of flexibility is possible but within the target's local parts only.

6 Within the 80 ns the 800 target-ligand conformations were generated via MD simulation for every SP, and only several from them are demonstrated in Fig. 4.

7 The Q552 of the 1st helix was attended by hydrophobic anchors of M_11+3dNb_ started from SP1b and SP2a; one of hydrophobic anchors contacted also with I548 (from the start SP2) and with L576 (from the start SP3); and anionic backbone units of the ligand polymeric chain also had an interactions with L576 (if the M_11+3dNb_ started from SP2).

8 The cationic ionisable R557 and R579 is one of the most active amino acid sites in binding the anionic units of polymeric chain.

9 The two N-terminal (Ile580 and Leu581) and two C-tail (Ser546 and Gly547) amino acid residues of every from three α-helixes were excluded from the consideration because of edge effects of the target cutoff (zones of a priori inadequacy between modelled and natural 3D structure of the target).

10 The monomeric units of polymeric chain or alicycles related to the pendant anchors.

11 Just as a combination of small amino acids is not protein, the biopolymer covalently cooperated a sequence of many amino acid residues, i.e. cooperatively accumulated their partial structures and functionalities.

12 For independent events (in the considered case, the mutations), the probability of the chance of all of two or more events occurring (intersection of the events) is the product of the probabilities.

13 The polymeric cooperation is the chemical linkage of variable small molecule monomers/units to entire polymeric macromolecule (for optimal structure—functionality). In fact the naturally biosynthesized polymeric cooperation is main route for molecular evolution of biological life. And analogical approaches in modern chemistry (the artificial anthropogenic synthesis) of polymers are "natural" post-biologic way toward novel materials, drugs, etc.

## References

[1] Arts EJ, Hazuda DJ (2012). HIV-1 antiretroviral drug therapy. Cold Spring Harb Perspect Med.

[2] Kumari G, Singh RK (2013). Anti-HIV drug development: structural features and limitations of present day drugs and future challenges in the successful HIV/AIDS treatment. Curr Pharm Des.

[3] Burstein ME, Serbin AV, Khakhulina TV, Alymova IV, Stotskaya LL, et al (1999) Inhibition of HIV-1 replication by newly developed adamantane-containing polyanionic agents. Antivir Res 41(3):135–144. PMID: 10320046 10.1016/s0166-3542(99)00006-6 10320046

[4] Timofeyev DI, Perminova NG, Kiseleva YY, Nekludov VV, Vatolin GY, et al (2003) HIV-inhibiting activity of polyanionic matrixes and based on them substances containing adamantine and norbornane pharmacophores. Antibiot Chemother (Russia) 48(5):7–15. PMID: 12968467 12968467

[5] Kiseleva YY, Perminova NG, Pliasunova OA, Timofeev DI, Serbin AV, et al (2005) Antiviral action of membranotropic compounds modified by adamantane and norbornene pharmacophores exerted on different HIV-1 strains. Mol Gen Mikrobiol Virusol (2):33–36. PMID: 15954475 15954475

[6] Bukrinskaya AG, Burshtain ME, Alikhanova OL, Ermakov IV, Kasyan LI, et al (2006) Polyanionic derivatives of norbornane, method of synthesis and based on them inhibitors of human immunodeficiency virus reproduction. Rus Pat 2281297. http://www.findpatent.ru/patent/228/2281297.html

[7] Serbin AV (2005) Design of bio-selective polymeric systems possessing the combined antiviral activity. D. Sci. Dissertation. http://www.lib.ua-ru.net/diss/cont/147035.html

[8] Serbin AV, Karaseva EN, Tsvetkov VB, Alikhanova OL, Rodionov IL (2010). Hybrid polymeric systems for nano-selective counter intervention in virus life cycle. Macromol Symp.

[9] Serbin AV, Veselovsky AV, Tsvetkov VB (2012). In vitro and in silico investigation of interferonogenic analogues of nucleic acids, artificially programmed to block the initial stages of HIV infection of cells. Appl Biochem Microbiol.

[10] Timofeev DI, Perminova NG, Serbin AV, Timofeev IV (2003) Membranotropic compounds and preparations affecting earlier stages of HIV-infection. Antibiot Chemother (Russia) 48(2):29–41. PMID: 12803048 12803048

[11] Lu M, Ji H, Shen S (1999). Subdomain folding and biological activity of the core structure from human immunodeficiency virus type 1 gp41: implications for viral membrane fusion. J Virol.

[12] Lu M, Stoller MO, Wang S, Liu J, Fagan MB (2001). Structural and functional analysis of interhelical interactions in the human immunodeficiency virus type 1 gp41 envelope glycoprotein by alanine-scanning mutagenesis. J Virol.

[13] Tan K, Liu JH, Wang JH, Shen S, Lu M (1997). Atomic structure of a thermostable subdomain of HIV-1 gp41. Proc Natl Acad Sci USA.

[14] Koshiba T, Chan DC (2003). The prefusogenic intermediate of HIV-1 gp41 contains exposed C-peptide regions. J Biol Chem.

[15] Wang X, Xiong W, Ma X, Wei M, Chen Y (2012). The conserved residue Arg46 in the N-terminal heptad repeat domain of HIV-1 gp41 is critical for viral fusion and entry. PLoS ONE.

[16] Tsvetkov VB, Serbin AV (2012). A novel view of modelling interactions between synthetic and biological polymers via docking. J Comput Aided Mol Des.

[17] Hertje M, Zhou M, Dietrich U (2010). Inhibition of HIV-1 entry: multiple keys to close the door. ChemMedChem.

[18] Cai L, Jiang S (2010). Development of peptide and small-molecule HIV-1 fusion inhibitors that target gp41. ChemMedChem.

[19] Zhou G, Wu D, Snyder B, Ptak RG, Kaur H, Gochin M (2011) Development of indole compounds as small molecule fusion inhibitors targeting HIV-1 glycoprotein-41. J Med Chem 54(24):7220–7231. doi:10.1021/jm200791z; PMID: 21928824 10.1021/jm200791z PMC3234170 21928824

[20] Wang H, Qi Z, Guo A, Mao Q, Lu H (2009). ADS-J1 Inhibits human immunodeficiency virus type 1 entry by interacting with the gp41 pocket region and blocking fusion-active gp41 core formation. Antimicrob Agents Chemother.

[21] Liu K, Lu H, Hou L, Qi Z, Teixeira C, et al (2008) Design, synthesis, and biological evaluation of N-carboxyphenylpyrrole derivatives as potent HIV fusion inhibitors targeting gp41. J Med Chem 51(24):7843–7854. PMCID: PMC2656571 10.1021/jm800869t PMC2656571 19053778

[22] Katritzky AR, Tala SR, Lu H, Vakulenko AV (2009). Design, synthesis, and structure-activity relationship of a novel series of 2-Aryl-5-(4-Oxo-3-phenethyl-2-thioxothiazolidinylidenemethyl)furans as HIV-1 entry inhibitors. J Med Chem.

[23] PDB 1AIK HIV gp41 core structure. doi:10.2210/pdb1aik/pdb

[24] Gasteiger J, Marsili M (1978). A new model for calculating atomic charges in molecules. Tetrahedron Lett.

[25] Case DA, Darden TA, Cheatham TE, Simmerling CL, Wang J (2006). AMBER 9.

[26] Hawkins GD, Cramer CJ, Truhlar DG (1996). Parametrized models of aqueous free energies of solvation based on pairwise descreening of solute atomic charges from a dielectric medium. J Phys Chem.

[27] Bashford D, Case DA (2000). Generalized born models of macromolecular solvation effects. Annu Rev Phys Chem.

[28] Wang J, Wolf RM, Caldwell JW, Kollamn PA, Case DA (2004). Development and testing of a general Amber force field. J Comput Chem.

[29] Ryckaert JP, Ciccotti G, Berendsen HJC (1977). Numerical integration of the cartesian equations of motion of a system with constraints: molecular dynamics of n-alkanes. J Comput Phys.

[30] Mark P, Nilsson L (2001). Structure and dynamics of the TIP3P, SPC, and SPC/E water models at 298 K. J Phys Chem A.

[31] Duan Y, Wu C, Chowdhury S, Lee MC, Xiong G (2003). A point-charge force field for molecular mechanics simulations of proteins based on condensed-phase quantum mechanical calculations. J Comput Chem.

[32] Darden T, York D, Pedersen L (1993). Particle mesh Ewald-an N·Log (N) method for Ewald sums in large systems. J Chem Phys.

[33] http://www.ks.uiuc.edu/Research/vmd/

[34] Knapp B, Lederer N, Omasits U, Schreiner W (2010). vmdICE: a Plug-in for rapid evaluation of molecular dynamics simulations using VMD. J Comput Chem.

[35] Onufriev A, Bashford D, Case DA (2000). Modification of the generalized born model suitable for macromolecules. J Phys Chem B.

[36] Onufriev A, Case DA, Bashford D (2002). Effective born radii in the generalized born approximation: the importance of being perfect. J Comput Chem.

[37] Weiser J, Shenkin PS, Still WC (1999). Approximate atomic surfaces from linear combinations of pairwise overlaps (LCPO). J Comput Chem.

[38] Tsvetkov VB (2012) Computational modeling the nano-bio-selective polymeric systems based on hetero- functional polyelectrolytes. Dissertation Ph.D. http://sp-department.ru/upload/iblock/1e4/1e40c59e60f6d0f4be66946010c8eebf.pdf

[39] Chan DC, Fass D, Berger JM, Kim PS (1997). Core structure of gp41 from the HIV envelope glyoprotein. Cell.

[40] Richardson JS (1981) The anatomy and taxonomy of proteins. Adv Prot Chem 34:67–339. doi:10.1016/S0065-3233(08)60520-3; PMID: 7020376 10.1016/s0065-3233(08)60520-3 7020376

[41] Mason JB, Arndt KM (2004) Coiled coil domains: stability, specificity, and biological implications. Chembiochem 5(2):170–176. doi:10.1002/cbic.200300781; PMID 14760737 10.1002/cbic.200300781 14760737

[42] Gallo SA, Puri A, Blumenthal R (2001). HIV-1 gp41 six-helix bundle formation occurs rapidly after the engagement of gp120 by CXCR4 in the HIV-1 Env-mediated fusion process. Biochemistry.

[43] Liechty WB, Kryscio DR, Slaughter BV, Peppas NA (2010). Polymers for drug delivery systems. Annu Rev Chem Biomol Eng.

[44] Kolocouris N, Foscolos GB, Kolocouris A, Marakos P, Pouli N (1994). Synthesis and antiviral activity evaluation of some aminoadamantane derivatives. J Med Chem.

[45] Kolocouris N, Kolocouris A, Foscolos GB, Fytas G, Neyts J et al (1996) Synthesis and antiviral activity evaluation of some aminoadamantane derivatives. 2. J Med Chem 39:3307–3318. PMID: 8765514 10.1021/jm950891z 8765514

[46] Frey G, Rits-Volloch S, Zhang XQ, Schooley RT, Chen B (2006). Small molecules that bind the inner core of gp41 and inhibit HIV envelope-mediated fusion. Proc Natl Acad Sci USA.

[47] Serbin AV, Karaseva EN, Alikhanova OL, Tsvetkov VB (2013) Drug resistance preventive antivirals based on nano-responsible poly-ligands. In Book: Worldwide research efforts in the fighting against microbial pathogen. From basic research to technological developments. Méndez-Vilas A (ed), Brown Walker Press, Boca Raton. ISBN-10:1-61233-636-1; ISBN-13: 978-1-61233-636-7, pp. 139–144. http://www.formatex.info/icar2012/ICAR2012-Proceedings.rar

[48] Tsvetkov V, Veselovski A, Serbin A (2011). Polymer-coupled systems for blocking the viral fusion 1. Modeling in silico the in vitro HIV-1 entry inhibitors. Antivir Res.

[49] Egorov Y, Serbin A, Kasyan L, Tarabara I, Alikhanova O (2006). Intramolecular alicyclic synergists for polyanionic antivirals. Antivir Res.

[50] Serbin A, Egorov Y, Alikhanova O (2007). Poly-cooperation of ionic and non-ionic antiviral vectors. Antivir Res.

[51] Serbin A, Karaseva E, Alikhanova O, Tsvetkov V (2011). Polymer-cooperative approach to multi-blocking the viruses. Antivir Res.

[52] De Clercq E (2006) Antiviral agents active against influenza A viruses. Nat Rev: Drug Discov 5:1015–1025. doi:10.1038/nrd2175. http://www.nature.com/nrd/journal/v5/n12/full/nrd2175.html 10.1038/nrd2175 PMC7097821 17139286

[53] Johnson VA, Calvez V, Günthard HF, Paredes R, et al (2013) Update of the drug resistance mutations in HIV-1: March 2013. Top Antivir Med 21(1):6–14. http://www.iasusa.org/sites/default/files/tam/21-1-6.pdf PMC6148891 23596273

