# Supplementary material for: Molecular dynamics modeling the synthetic and biological polymers interactions pre-studied via docking: Anchors modified polyanions interference with the HIV-1 fusion mediator
Source: J Comput Aided Mol Des. 2014 May 27;28(6):647–73. doi: 10.1007/s10822-014-9749-8 (PMC4050303; doi:10.1007/s10822-014-9749-8)
Supplement: Supplementary file 1 — Supplementary material 1 (DOC 60 kb) [file 10822_2014_9749_MOESM1_ESM.doc]

***Supplementary material 1***

***Some definitions applied in this article for the H-bonds analysis***

*The H-bond formation* was accepted as the fact for the group atoms A_i_-H A_j_, where the A_i_ and A_j_ were atoms of oxygen (O) or nitrogen (N), if the criteria indicated in the experimental part (the donor-acceptor distance of 3.5 Å and an angle cutoff of 30 degrees) were realized.

Such facts were monitored individually for all N and O atoms of the target and ligands for every MD simulated conformation (single snapshot) followed by accumulation and analysis of this data within full MD simulated time/trajectory (80 ns, 800 snapshots).

*Quantity of H-bonds within a single snapshot (****Q_Hb_****)* was defined as a sum of the H-bonds formation facts recorded for this concrete snapshot (for the relevant moment of simulated time). This quantity could be calculated in account for full target-ligand network of N/O atoms or for selected sub-objects separately (separately for target, target’s α-helixes or cavity levels L1-3, amino acids, the acids’ individual N/O atoms, and/or for ligand, its structural groups or chosen individual atoms), depending on purposes of the analysis. For the single group of A_i_-H A_j_ atoms the *Q_Hb_* = 0 (when the H-bond was not formed) or *Q_Hb_* = 1 (if the H-bond was taken place within the concrete snapshot). For an assemblage of atoms selected for analysis, the *Q_Hb_* may be more than 1, taking on integer number values, but not more than quantity of the analyzed H-bond able groups of atoms.

*Degree of H-bonds formation (H-bonding) statistically averaged for a time interval of MD simulation (****D_Hb_****).*

The ***D_Hb_*** value for individual group of some A_i_-H A_j_ atoms was defined as the quotient calculated by the ratio of (quantity of snapshots where the *Q_Hb_* = 1 for this pair of atoms) to (full quantity of MD simulated snapshots, that equal 800 in case of simulated 80 ns or the proportionally less in case of shorter time interval analyzed). For every single H-bond able atom the *D_Hb_* could take on values of 0 ≤ *D_Hb_* ≤ 1, including a fractional numbers. Therefore, for ensembles of analyzed atoms the *D_Hb_* could be more than 0 or 1, taking both integer and fractional number values (in contrast with *Q_Hb_* allowing integer numbers only).

*The D_Hb_ and Q_Hb_ are in the relation (3):*

*D_Hb_ = Σ^m^ Q_Hb_^i^/m* (3)

where the *Q_Hb_^i^* – amount of H-bonds for analyzed pool of N/O atoms in *i’th* target/ligand (or target + ligand) conformation (in *i'th* single snapshot), *m –* number of the MD generated conformations (snapshots) taken into account for analysis, and the *Σ^m^ Q_Hb_^i^ –* total amount of the H-bonds within the *m* snapshots.

*I.g., the D_Hb_ is Q_Hb_ statistically “m snapshots-averaged” (for considered number m of MD generated conformations).*

After the calculations for all possible pairs of N and O atoms, these data sets were treated for quantitative estimation of summarized amounts or degrees of H-bonds as well as for analysis of separate contributions of various sub-structural components of ligands and target to the H-bond network.

To differentiate various analytically relevant series of H-bonds we introduced the following definitions for H-bonds classification, depending on the H-bond forming atoms belonging:

**M/M** – H-bond between atoms from main chain (the polypeptide backbone) of target;

**M/S** – H-bond between atom(s) of the main chain and atom(s) of an amino acid residue side chain or

**S/M** – H-bond between atom(s) of an amino acid residue side chain and atom(s) of the main chain;

**S/S** – H-bond between atoms from the side chains of target

**M/L** – H-bond between atom(s) from target’s main chain and atom(s) of ligand

**S/L** – H-bond between atom(s) from a side chain of target and atom(s) of ligand
