# Supplementary material for: Molecular dynamics modeling the synthetic and biological polymers interactions pre-studied via docking: Anchors modified polyanions interference with the HIV-1 fusion mediator
Source: J Comput Aided Mol Des. 2014 May 27;28(6):647–73. doi: 10.1007/s10822-014-9749-8 (PMC4050303; doi:10.1007/s10822-014-9749-8)
Supplement: Supplementary file 2 — Supplementary material 2 (DOC 127 kb) [file 10822_2014_9749_MOESM2_ESM.doc]

***Supplementary material 2***

**
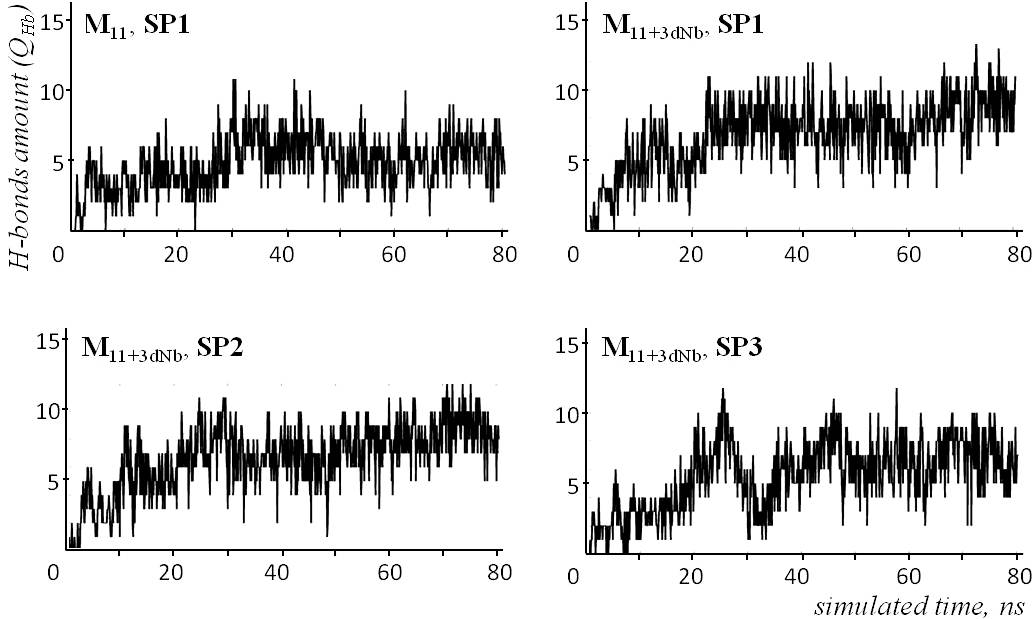
**

**Fig. 6**  **Dynamics of H-bonds formation** between the target and ligands from various starting positions (SP1, SP2, and SP3)
