# Supplementary material for: Molecular dynamics modeling the synthetic and biological polymers interactions pre-studied via docking: Anchors modified polyanions interference with the HIV-1 fusion mediator
Source: J Comput Aided Mol Des. 2014 May 27;28(6):647–73. doi: 10.1007/s10822-014-9749-8 (PMC4050303; doi:10.1007/s10822-014-9749-8)
Supplement: Supplementary file 3 — Supplementary material 3 (DOC 600 kb) [file 10822_2014_9749_MOESM3_ESM.doc]

***Supplementary material 3***


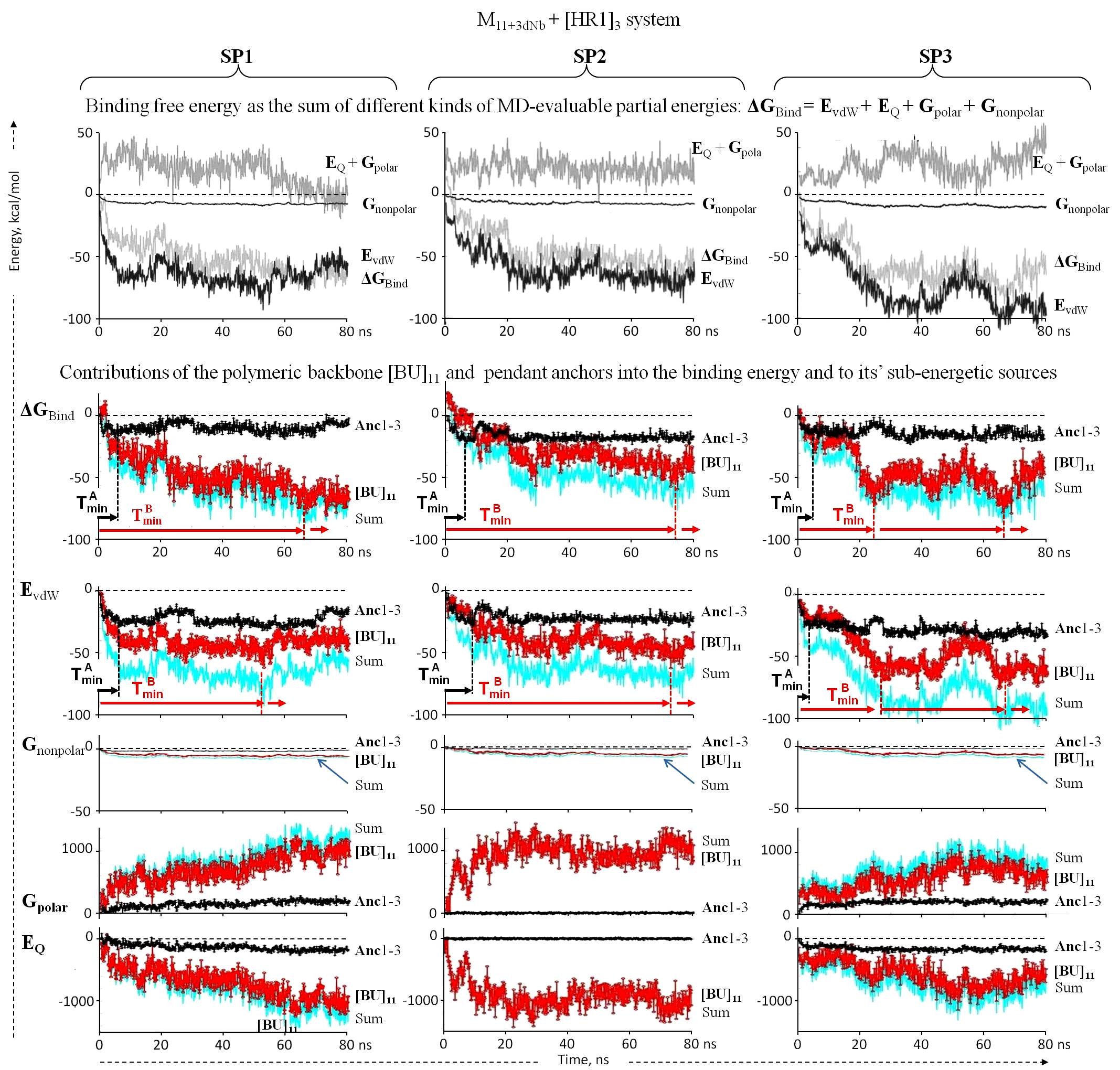


**Fig. 7** **M_11+3dNb_ + [NHR]_3_ binding energy (ΔG_Bind_) dynamics** from various starting positions (SP1, SP2 and SP3) in view of various kinds of MD-evaluable sub-energetic sources: van der Waals forces energy (**E_vdW_**), Coulomb energy (**E_Q_**), polar, the electrostatic (**G_polar_**), and nonpolar (**G_nonpolar_**) parts of solvation energy; as well as separated contributions of polymeric chain backbone ([-BU-]_11_) and pendant anchors (Anc1-3) to the full ligand binding energy, and to the all involved in the ΔG**_Bind_** sub-energies of the considered kinds.

*Note:* **T^A^_min_** *and* **T^B^_min_** *- time periods for achievement of minimum of the partial contribution to ΔG****_Bind_*** *(or to E_vdW_, for example) by the pendant anchors and by the polymeric chain, respectively.*
