# Supplementary material for: Molecular dynamics modeling the synthetic and biological polymers interactions pre-studied via docking: Anchors modified polyanions interference with the HIV-1 fusion mediator
Source: J Comput Aided Mol Des. 2014 May 27;28(6):647–73. doi: 10.1007/s10822-014-9749-8 (PMC4050303; doi:10.1007/s10822-014-9749-8)
Supplement: Supplementary file 4 — Supplementary material 4 (DOC 51 kb) [file 10822_2014_9749_MOESM4_ESM.doc]

***Supplementary material 4***

***Cooperative effects by example of the M_11+3dNb_ + [HR1]_3_ system from the SP1 start***

*(step-by-step analysis of the MD simulated snapshots in dynamics of 0.1 ns intervals)*

Initially (by the 0,1 ns, Fig. 4a) the Anc2 bound with target in L1 pocket (near Leu568 of 1^st^ helix), inducing (through the covalent bridge) next contacts of close acidic fragment of polymeric chain with Lys574 (of 1^st^ helix at L1). The contacted with Lys574 fragment BU, being coupled in the [BU]_11_ chain with subsequent acidic units, involved next one by one units in interactions with target too. Then the Anc2 was in regular contacting not only with the Leu568 but also with hydrophobic Trp571 (of 1^st^ helix at L1) and Gln567 (of 1^st^ helix at the L1 edge toward L2), cowering the L1 pocket in cooperation with acidic chain units, which added active contacts with the basic Lys574 and more basic guanidine group of Arg579 (of 1^st^ helix at L1), as well as with Ala578 and H-bond able Gln575 (of 1^st^ helix at L1) (Fig. 4a and Fig. 5 - SP1b). These multipoint interactions maintained binding network developed enough for stabilization of considerable part of ligand molecule in locus of the L1 pocket.

Almost simultaneously from the mentioned start (by 2.4 ns, Fig. 4a), other anchor (the Anc1) begun to bind the target also, but at another, the L2 cavity near Leu556 (of 1^st^ helix). This contact entrained the linked (by Anc1 bridge) the acidic –BU– and following polymeric chain of subsequently coupled acidic units. This chain motif became involved in interactions with target also, predominantly through contacts of carboxylic groups with guanidine group of Arg557, as well as with Leu556 (of 1^st^ helix). After that the Arg557 played role of one of most active centers for multiple reversible binding – dissociation – rebinding of oxygen atoms from sequence of polymeric chain (see Fig. 5 – SP1b and section 4.5). In the meantime, the neighbor tail of polyacidic chain found stabilization at next, the L3 cavity via multipoint contacts with Gln550, Gln551 (of 3^rd^ helix), Asn553 and Gln550 (of 1^st^ helix), that caused a centipede-like removing (see section 4.5) of some part of polyacidic motifs from L2 toward the L3, as well as dissociation of Anc1 from L2 and it migration toward L3 too. Within the L3 Anc1 met Val549 (of 1^st^ helix) as a partner for stable hydrophobic contact in additional connections with surrounding Gln550, Gln552 and Asn553 (of 1^st^ helix). Simultaneously the acidophilic Arg577 in L2 was filled by binding with next portion of acidic units of the multi-unit polymeric chain of ligand. This network of reversible multipoint contacts between various sites of target and different components of ligand molecule was in dynamic balance of alterations, keeping mostly synergism of cooperation between the BUs and anchors in binding the target. However, some local manifestations of mutual competition between the ligand components were observed also. For instance, this could be noted in relation to the interactions at L3 cavity, where the hydrophobic Val549, high attractive to Anc2, was closely surrounded by hydrophilic and H-bond able residues of Gln550, Gln552 and Asn553 attractive for BUs. It provoked a spatial competition between the Anc1 and acidic polymeric chain motifs for occupation of the cavity at L3 of target. Reversible displacement of Anc1 by the units of chain was also promoted by geometrical fluctuations of closely (near L2) spaced part of ligand’s chain motifs in interactions with Arg557. Swelling and bending of polymeric backbone in the locus of contacts with Arg557 induced successive displacements of back-to-back co-linked chain units. This chain-mediated translocations was able (in view of rigidity within short sections of polymeric chain) to push the back-to-back linked BUs into the L3 cavity, replacing the Anc1 from the same area. But this competition was in reversible balance, and intensity of the Gln550 and Asn553 attendance by –BU– species of chain was comparable with attendance by Anc1(Fig. 5 – SP1b)

In contrast with Anc1/Anc2, the initial contact between Anc3 and target (near Leu581 of 1^st^ helix at L1 outskirts) was not very stable, allowing expressed fluctuations and periodic loss of this contact. However, an ability of Anc3 to move away from target was found to be strongly restricted by the bridge between Anc3 and acidic unit of polymeric chain, many parts of which (in cooperation with Anc1 and Anc2) were in state of multipoint binding with target from L1 to L3 areas. This linkage of units in polymeric molecule and the polymer-cooperated interaction of ligand with target resulted in the limitation of allowed distance for Anc3 moving away from the target not farther than 1 nm (Fig. 4a). So the Anc3 (being on a flexible but short leash through bridge and polymeric chain) regularly returned to the target renewing contacts with one.
